# Seizure-mediated iron accumulation and dysregulated iron metabolism after status epilepticus and in temporal lobe epilepsy

**DOI:** 10.1007/s00401-021-02348-6

**Published:** 2021-07-22

**Authors:** Till S. Zimmer, Bastian David, Diede W. M. Broekaart, Martin Schidlowski, Gabriele Ruffolo, Anatoly Korotkov, Nicole N. van der Wel, Peter C. van Rijen, Angelika Mühlebner, Wim van Hecke, Johannes C. Baayen, Sander Idema, Liesbeth François, Jonathan van Eyll, Stefanie Dedeurwaerdere, Helmut W. Kessels, Rainer Surges, Theodor Rüber, Jan A. Gorter, James D. Mills, Erwin A. van Vliet, Eleonora Aronica

**Affiliations:** 1 Department of (Neuro)Pathology, Amsterdam UMC, University of Amsterdam, Amsterdam Neuroscience, Meibergdreef 9, 1105 AZ Amsterdam, The Netherlands; 2 Department of Epileptology, University Hospital Bonn, Bonn, Germany; 3 German Center for Neurodegenerative Diseases (DZNE), Bonn, Germany; 4 Laboratory affiliated to Istituto Pasteur Italia, Department of Physiology and Pharmacology, University of Rome Sapienza, Rome, Italy; 5 Department Cell Biology and Histology, Amsterdam UMC, University of Amsterdam, Amsterdam, The Netherlands; 6 Department Electron Microscopy Center Amsterdam, Amsterdam UMC, University of Amsterdam, Amsterdam, The Netherlands; 7 Department of Neurosurgery, Brain Centre, Rudolf Magnus Institute for Neuroscience, University Medical Center Utrecht, Utrecht, The Netherlands; 8 Department of Pathology, University Medical Center Utrecht, Utrecht, The Netherlands; 9 Department of Neurosurgery, Amsterdam Neuroscience, Amsterdam UMC, Vrije Universiteit Amsterdam, Amsterdam, The Netherlands; 10 Neurosciences Therapeutic Area, UCB Pharma, Braine-l’Alleud, Belgium; 11 Center for Neuroscience, Swammerdam Institute for Life Sciences, University of Amsterdam, Amsterdam, The Netherlands; 12 Department of Clinical and Experimental Epilepsy, UCL, London, UK; 13 Chalfont Centre for Epilepsy, Chalfont St Peter, UK; 14 Stichting Epilepsie Instellingen Nederland (SEIN), Heemstede, The Netherlands

**Keywords:** Iron, Glutathione metabolism, Status epilepticus, Temporal lobe epilepsy with hippocampal sclerosis, Astrocytes

## Abstract

Neuronal dysfunction due to iron accumulation in conjunction with reactive oxygen species (ROS) could represent an important, yet underappreciated, component of the epileptogenic process. However, to date, alterations in iron metabolism in the epileptogenic brain have not been addressed in detail. Iron-related neuropathology and antioxidant metabolic processes were investigated in resected brain tissue from patients with temporal lobe epilepsy and hippocampal sclerosis (TLE-HS), post-mortem brain tissue from patients who died after status epilepticus (SE) as well as brain tissue from the electrically induced SE rat model of TLE. Magnetic susceptibility of the presumed seizure-onset zone from three patients with focal epilepsy was compared during and after seizure activity. Finally, the cellular effects of iron overload were studied in vitro using an acute mouse hippocampal slice preparation and cultured human fetal astrocytes. While iron-accumulating neurons had a pyknotic morphology, astrocytes appeared to acquire iron-sequestrating capacity as indicated by prominent ferritin expression and iron retention in the hippocampus of patients with SE or TLE. Interictal to postictal comparison revealed increased magnetic susceptibility in the seizure-onset zone of epilepsy patients. Post-SE rats had consistently higher hippocampal iron levels during the acute and chronic phase (when spontaneous recurrent seizures are evident). In vitro, in acute slices that were exposed to iron, neurons readily took up iron, which was exacerbated by induced epileptiform activity. Human astrocyte cultures challenged with iron and ROS increased their antioxidant and iron-binding capacity, but simultaneously developed a pro-inflammatory phenotype upon chronic exposure. These data suggest that seizure-mediated, chronic neuronal iron uptake might play a role in neuronal dysfunction/loss in TLE-HS. On the other hand, astrocytes sequester iron, specifically in chronic epilepsy. This function might transform astrocytes into a highly resistant, pro-inflammatory phenotype potentially contributing to pro-epileptogenic inflammatory processes.

**Supplementary Information:**

The online version contains supplementary material available at 10.1007/s00401-021-02348-6.

## Introduction

Epilepsy is a common neurological disease that is characterized by “an enduring predisposition to generate epileptic seizures, and by the neurobiologic, cognitive, psychological, and social consequences of this condition” [18, 19]. Epileptogenesis describes the pathogenic process by which physiological and structural changes in the brain are induced, leading to increased seizure susceptibility and enhanced likelihood of spontaneous recurrent seizures (SRS) to occur [61]. A major pathogenic mechanism of epileptogenesis in epilepsies of various etiology is oxidative stress (OS) [58, 75, 81].

OS is defined as an imbalance between the antioxidant capacity of a cell or tissue and the generation of reactive oxygen species (ROS) with a shift towards the latter leading to cell damage or even cell death. During acute seizure activity, excessive ROS are generated due to i.e. mitochondrial dysfunction and/or increased activity of nicotinamide adenine dinucleotide phosphate (NADPH) oxidase [37, 69, 81]. Targeting OS by providing ROS scavenging antioxidants or by boosting endogenous antioxidant systems, specifically the activity of the antioxidant transcription factor nuclear factor erythroid 2 like 2 (Nrf-2), was shown to have a beneficial disease-modifying effect in models of acquired epilepsy, delaying onset and progression of seizures [45, 58, 74, 76]. These effects were likely facilitated by proteins involved in the production and recycling of the primary intracellular antioxidant glutathione [7].

In this context, recent evidence from congenital epilepsies due to focal cortical dysplasia and tuberous sclerosis complex indicates that OS and iron metabolism are tightly linked and could act synergistically to exacerbate cell dysfunction and damage in epilepsy [97]. Accordingly, high concentrations of unbound Fe^2+^ iron could act as catalyst in the Haber–Weiss and Fenton reactions to enhance the potency of hydrogen peroxide from cellular processes to more reactive ROS like the hydroxyl radical or hydroxide ion, thereby facilitating cell dysfunction or death [22, 82]. Besides congenital epilepsies, intracerebral hemorrhages resulting from traumatic brain injury (TBI) and sequential post-traumatic epilepsy are characterized by brain iron deposition and intracortical iron injections evoke seizures and epilepsy in experimental models [1, 35, 40, 42, 50, 71, 77, 89]. Additionally, independent of large-scale hemorrhages, dysfunction of the BBB is a hallmark of a variety of epileptogenic pathologies in which iron concentrations could increase locally by non-specific leakage of iron-rich blood components into the brain parenchyma and subsequent iron release [20, 85]. In line with this, previous microarray studies of brain tissue from low-grade epilepsy-associated tumors and a rat model of acquired epilepsy point towards a critical role of iron metabolism in epilepsy [3, 24]. However, up to now, alterations in iron metabolism and its consequences have not been assessed in detail in brain tissue from patients with temporal lobe epilepsy (TLE).

This study aims to investigate (1) if iron accumulation and iron metabolism are altered after *status epilepticus* (SE) and in TLE per se in conjunction with OS and antioxidant capacity, (2) which cell types are involved in iron regulation and (3) whether iron has ictogenic effects and could be involved in epileptogenesis. To this end, we evaluated OS and iron metabolism in post-mortem brain tissue of patients who died after SE and in surgically resected brain tissue from patients suffering from intractable TLE with hippocampal sclerosis (TLE-HS) to elucidate the effects of acute seizure activity and chronic epilepsy in iron metabolism, respectively. These findings were further validated by assessing the transcriptome of a cohort of TLE-HS hippocampal tissue. Furthermore, by employing quantitative susceptibility mapping (QSM) of three patients with focal epilepsies, spatial and temporal changes in iron in the seizure-onset zone were assessed. Consequently, iron overload and affected cell types were studied during different stages of epileptogenesis in the electrical post-SE model of TLE. To elucidate the cellular response to iron-mediated OS hippocampal brain slices and human fetal astrocytes were stimulated with iron in vitro.

## Materials and methods

### Subjects

Surgical and post-mortem brain tissue included in this study was obtained from the archives of the departments of Neuropathology of the Amsterdam UMC, (Amsterdam, The Netherlands) and the UMC Utrecht (Utrecht, The Netherlands). Hippocampal brain samples were obtained from patients undergoing surgery for intractable epilepsy and diagnosed with TLE-HS. All cases were reviewed independently by two neuropathologists. The classification of HS was determined as described by the International League against Epilepsy [4]. The hippocampus and cortex of age-matched controls without a history of seizures or other neurological diseases and cortical brain tissue from patients who died after SE, stroke or traumatic brain injury (TBI) was obtained at autopsy. All autopsies were performed within 24 h after death. Clinical details of patient cohorts and number of samples for experiments used in this study are summarized in Tables 1, 2, 3 and 4 and Online resource 7. Tissue was obtained with informed consent for the use in research and access to medical records in accordance with the Declaration of Helsinki and the Amsterdam UMC Research Code provided by the Medical Ethics Committee.

**Table 1:** Summary of experimental groups

Experimental group	Diagnosis	*N*	Median age in years (range)	Gender (m/f)
RNA	Autopsy control	10	71 (25–79)	6/4
	TLE-HS	13	42 (29–66)	6/7
Protein	Autopsy control	6	48 (35–63)	3/3
	TLE-HS	9	38 (6–66)	4/5
CSF	CSF control	6	61 (38–70)	3/3
	TLE-HS	8	43 (32–57)	6/2
IHC	Autopsy control	5	39 (25–67)	1/4
	Status epilepticus	5	79 (31–87)	3/2
	TLE-HS	6	36 (29–60)	3/3
RNA sequencing	Autopsy control	11	62 (30–72)	7/4
	TLE-HS	33	44 (32–62)	22/11

*TLE-HS* temporal lobe epilepsy with hippocampal sclerosis, *m* male, *f* female, *CSF* cerebrospinal fluid, *IHC* immunohistochemistry, *N* number of cases

Mann–Whitney *U* test of age-matched status: RNA *p* = 0.0586; Protein *p* = 0.3156; CSF *p* = 0.0526; IHC *p* = 0.9307; RNA sequencing *p* = 0.0526

**Table 2:** Clinical information of autopsy control, cerebrospinal fluid control and status epilepticus patients

Group	Case ID		Age	Gender	Cause of death/reason for CSF sampling	Post-mortem delay (h)	Neuropathological conclusion	SE to death delay
Autopsy control	4	a	71	f	bronchopneumonia	10	normal, mild hypoxia	N/A
Autopsy control	5	a	70	f	Peritonitis	15	Normal	N/A
Autopsy control	6	a, e	30	m	Myocardial infarction	8	Normal	N/A
Autopsy control	7	a	76	m	Myocardial infarction	9	Normal, mild hypoxia	N/A
Autopsy control	8	a	72	m	Respiratory insufficiency	11	Normal	N/A
Autopsy control	9	a, d	25	f	Arrhythmia	7	Normal	N/A
Autopsy control	10	a	79	m	Respiratory insufficiency	12	Normal	N/A
Autopsy control	11	a, e	69	m	Bronchopneumonia	10	Normal	N/A
Autopsy control	12	a, b, d	35	f	Myocarditis	9	Normal	N/A
Autopsy control	13	a	75	m	Bronchopneumonia	11	Normal	N/A
Autopsy control	14	b, e	62	m	Bronchopneumonia	9	Normal, mild hypoxia	N/A
Autopsy control	15	b, e	63	f	Pulmonary embolism	11	Normal	N/A
Autopsy control	16	b	35	f	Myocarditis	8	Normal	N/A
Autopsy control	17	b, e	49	m	Bronchopneumonia	10	Normal	N/A
Autopsy control	18	b, e	47	m	Cardiac tamponade	8	Normal	N/A
Autopsy control	19	b	38	f	Pneumothorax	11	Normal	N/A
Autopsy control	20	c	51	m	Chronic headache	N/A	Normal	N/A
Autopsy control	21	c	60	m	Facial hypoesthesia	N/A	Normal	N/A
Autopsy control	22	c	61	f	Chronic headache	N/A	Normal	N/A
Autopsy control	23	c	70	m	Cranial nerve palsy	N/A	Normal	N/A
Autopsy control	24	c	68	f	Myelopathy	N/A	Normal	N/A
Autopsy control	25	c	38	f	Suspected Guillain-Barré syndrome	N/A	Normal	N/A
Autopsy control	26	d	39	f	Cardiorespiratory failure	10	Normal	N/A
Autopsy control	27	d	67	m	Cardiorespiratory failure	9	Normal	N/A
Autopsy control	28	d	25	f	Cardiorespiratory failure	12	Normal	N/A
Autopsy control	29	d	35	f	Myocarditis	10	Normal	N/A
Autopsy control	30	d	45	f	Arrhythmia	7	Normal	N/A
Status epilepticus	31	d	58	m	Frontal lobe infarction, thrombosis	10	Non-convulsive SE	3 d
Status epilepticus	32	d	79	f	Myocardial infarction	8	Non-convulsive SE	7 w
Status epilepticus	33	d	81	m	Pneumonia	14	Convulsive SE	0 d
Status epilepticus	34	d	31	m	Astrocytoma (WHO III)	9	Convulsive SE	5 d
Status epilepticus	35	d	87	f	Aneurysm arteria cerebri media	7	Convulsive SE	10 d
Autopsy control	36	e	63	f	Arrhythmia—ventricular fibrillation	8	Normal, mild Hypoxia	N/A
Autopsy control	37	e	67	f	Multiple organ failure	10	Normal	N/A
Autopsy control	38	e	34	m	Arrhythmia—cardiomyopathy	7	Normal, mild hypoxia	N/A
Autopsy control	39	e	72	m	Bronchopneumonia	11	Normal	N/A
Autopsy control	40	e	44	f	Bronchopneumonia	9	Normal	N/A

Letters in case ID indicate applied technique to the respective sample: a = qPCR, b = protein analyses, c = cerebrospinal fluid (CSF) analyses, d = immunohistochemistry, e = RNA sequencing

*N/A* not applicable, *m* male, *f* female, *y* years, *d* days, *w* weeks, *h* hours

**Table 3:** Clinical information of TLE-HS patients who underwent surgical resection for epilepsy therapy

Case ID		Age (y)	Gender	TLE-HS type	Duration epilepsy (y)	Seizures/month	Seizure type	ASD	Etiology/comments
1	a	36	f	1	32	6	FA, FB/TC	LMT	N/A
2	a, b	66	f	1	60	8	FIA	CBZ, CLB	N/A
3	a	50	m	1	22	10	FIA	LMT, CBZ	N/A
4	a	42	f	2	28	1	FIA, FB/TC	PHT, OXC	N/A
5	a	29	m	1	11	15	FIA, FB/TC	CBZ	N/A
6	a	29	f	1	16	32	FIA, FB/TC	LMT, TPM	History of febrile convulsions
7	a, c	57	f	1	1	1	FIA	CNP	Possible history of febrile convulsions
8	a	39	m	1	5	32	FIA	CBZ	N/A
9	a	43	m	1	40	8	FIA, FB/TC	LEV, LMT	N/A
10	a	31	f	2	10	4	FIA	CBZ, CLB	N/A
11	a	49	m	1	41	2	FIA	LEV, CBZ, CLB	Possible history of CNS infection
12	a	44	f	2	22	3	FIA	CBZ, LMT	History of CNS infection
13	a	38	m	2	10	6	FIA	CBZ	N/A
14	b	35	f	1	33	150	FIA	CBZ, CLB	N/A
15	b	53	m	1	50	60	FIA	LMT, CBZ	N/A
16	b	51	m	1	51	200	FIA, FB/TC	LEV	N/A
17	b	19	f	1	12	17	FIA	TPM	N/A
18	b	6	f	1	5	20	FIA	LMT	N/A
19	b	38	f	1	36	40	FIA	LCS, LMT, CLB	N/A
20	b	14	m	1	7	17	FIA	LEV, CBZ	Posterior reversible encephalopathy (PRES) syndrome
21	b	39	m	1	5	4	FIA, FB/TC	LCS, CLB, CNP	N/A
22	c	32	f	1	17	4	FIA	CBZ, LEV	N/A
23	c	55	m	3	7	3	FA, FIA	CBZ	N/A
24	c	37	m	1	20	15–20	FIA	CBZ	N/A
25	c	38	m	2	10	6	FIA	CBZ	N/A
26	c	57	m	1	7	> 20	FIA	LMT	N/A
27	c	43	m	1	43	1	FIA	LMT, CBZ	N/A
28	c	42	m	1	25	20	FIA	CBZ, PGB	N/A
29	d	50	f	1	40	30	FA, FB/TC	CBZ, LEV, PHT	N/A
30	d	41	f	1	29	8	FIA	CBZ, TPM	N/A
31	d	31	m	1	28	20	FIA	CBZ	Adverse events following smallpox vaccination
32	d	60	f	1	54	3	FIA, FB/TC	CBZ, LEV	Possible history of CNS infection
33	d	29	m	1	13	36	FIA	CBZ, CLB	History of febrile convulsions
34	d	29	m	1	14	36	FIA	CBZ, CLB	History of febrile convulsions
35	d	29	m	3	25	12	FIA	CBZ, CLB	Possible history of CNS infection
36	e	41	m	1	26	4	FIA	LEV, LMT, CLB	N/A
37	e	56	m	1	52	25	FIA	CBZ, LEV	N/A
38	e	38	f	2	17	7	FIA	LMT, CLB	N/A
39	e	60	f	1	54	3	FIA, FB/TC	CBZ, LEV	N/A
40	e	36	f	1	32	6	FA, FB/TC	LMT	N/A
41	e	41	m	1	30	30	FIA	CBZ, CLB	N/A
42	e	42	f	2	26	1	FIA, FB/TC	PHT, OXC	N/A
43	e	55	m	3	7	3	FIA	CBZ	N/A
44	e	39	m	1	29	3	FIA, FB/TC	LMT, OXC	N/A
45	e	49	m	1	39	2	FIA	LEV, CBZ, CLB	N/A
46	e	38	m	2	8	6	FIA	CBZ	N/A
47	e	43	m	1	39	8	FIA, FB/TC	LEV, LMT	N/A
48	e	49	m	1	33	5	FIA, FB/TC	CBZ	N/A
49	e	54	f	1	33	20	FIA, FB/TC	VPA, OXC	N/A
50	e	43	m	1	19	5	FIA, FB/TC	CBZ, VPA, LMT	N/A
51	e	39	f	1	23	5	FIA	ZNS, LMT, CLB	N/A
52	e	43	m	1	37	5	FIA	OXC, LEV	N/A
53	e	32	f	1	14	5	FIA	CBZ, LEV, CLB	N/A
54	e	62	m	1	38	6	FIA, FB/TC	PHT, LMT, PB	N/A
55	e	47	f	1	12	3	FIA, FB/TC	LEV, LCS	N/A
56	e	53	m	1	50	60	FIA	LMT, CLB	N/A
57	e	41	m	1	39	20	FIA	CBZ, LEV	N/A
58	e	60	m	1	46	20	FIA	LMT, LEV	N/A
59	e	51	m	1	51	200	FIA, FB/TC	LEV, LOR	N/A
60	e	38	f	1	36	40	FIA	LCS, LMT, CLB	N/A
61	e	38	m	1	26	4	FIA	OXC, TPM	History of febrile convulsions
62	e	46	f	1	36	4	FIA, FB/TC	LMT, OXC, CLB	N/A
63	e	44	m	1	11	13	FIA	LMT	N/A
64	e	39	m	1	5	4	FIA, FB/TC	LCS, CLB, CNP	N/A
65	e	58	m	1	40	4	FIA, SE	CBZ, LCS	N/A
66	e	58	f	1	52	6	FIA, FB/TC	CBZ, LMT, PGB, CLB	N/A
67	e	58	m	1	25	30	FIA	PGB, CLB, PER	N/A
68	e	53	m	2	27	30	FIA	VPA, CBZ, CLB, LEV	N/A

Letters in case ID indicate applied technique to the respective sample: a = qPCR; b = protein analyses; c = cerebrospinal fluid (CSF) analyses; d = immunohistochemistry; e = RNA sequencing

*ASD* antiseizure drug, *m* male, *f* female, *y* years, *FA* focal aware, *FIA* focal impaired awareness, *FB/TC* focal bilateral/tonic clonic, *BRV* brivaracetam, *CBZ* carbamazepine, *CLB* clobazam, *CNP* clonazepam, *ESM* ethosuximide, *LCS* lacosamide, *LEV* levetiracetam, *LMT* lamotrigine, *LOR* lorazepam, *OXC* oxcarbazepine, *PB* phenobarbital, *PER* perampanel, *PGB* pregabalin, *PHT* phenytoin, *TPM* topiramate, *VPA* valproate, *ZNS* zonisamide, *N/A* not applicable, *CNS* central nervous system

HS typing performed according to the ILAE consensus classification on hippocampal sclerosis in TLE [4].

**Table 4:** Summary of clinical information of quantitative susceptibility mapping cohort

	Condition	Duration epilepsy (y)	Seizures/month	Seizure type	pSOZ	ASD
Patient #1	Non-lesional	12	3–4	FB/TC	Left temporal lobe	LEV, LMT, ECB
Patient #2	Left mesio-temporal GG	12	0.25	FB/TC	Left temporal lobe	BRI, LMT
Patient #3	FCD 2b	21	30–60	FIA	Right frontal lobe	OXC, ZNS, CBD

*ASD* antiseizure drug, *FCD 2b* focal cortical dysplasia type 2b, *GG* ganglioglioma, *FB/TC* focal to bilateral tonic–clonic, *pSOZ* presumed seizure-onset zone, *FIA* focal impaired awareness, *BRI* Brivaracetam, *CBD* Cannabidiol, *ECB* Eslicarbazepine, *LEV* Levetiracetam, *LMT* Lamotrigine, *OXC* Oxcarbazepine, *ZNS* Zonisamide, *y* years

### Immunohistochemistry on paraffin-embedded brain tissue

Human brain tissue was fixed in 10% buffered formalin and embedded in paraffin. Paraffin-embedded tissue was sectioned at 6 µm, mounted on pre-coated glass slides (Star Frost, Waldemar Knittel Glasbearbeitungs, Braunschweig, Germany) and processed for immunohistochemical stainings. Sections were deparaffinated in xylene, rinsed in ethanol (100%, 95%, 70%) and incubated for 20 min in 0.3% H_2_O_2_ diluted in methanol to block endogenous peroxidase activity. Antigen retrieval was performed using a pressure cooker in 0.01 M sodium citrate buffer (pH 6.0) at 121 °C for 10 min. Slides were washed with phosphate-buffered saline (PBS; 0.1 M, pH 7.4) and incubated overnight with primary antibodies against phosphorylated 4-hydroxynonenal (4-HNE; rabbit polyclonal, Abcam, Cambridge, UK; 1:500), heme oxygenase 1 (HO-1; rabbit polyclonal, Abcam, Cambridge, UK; 1:200), DMT-1 (SLC11A2; rabbit polyclonal, Proteintech, Rosemont, IL, USA; 1:500), anti-horse spleen ferritin (against rat tissue; polyclonal rabbit, Sigma-Aldrich, St. Louis, MO, USA; 1:2000), anti-human ferritin (rabbit polyclonal, DAKO, Glostrup, Denmark; 1:750), peroxiredoxin 6 (PRX6; rabbit polyclonal, Merck-Millipore, Burlington, MA, USA; 1:100) or TFRC (rabbit polyclonal, Proteintech, Rosemont, IL, USA; 1:200) in antibody diluent (VWR International, Radnor, PA, USA) at 4 °C. Thereafter, slides were washed in PBS and then stained with a polymer-based horse radish peroxidase (HRP) immunohistochemistry detection kit (Brightvision plus kit, ImmunoLogic, Duiven, The Netherlands) according to the manufacturer’s instructions. After washing in PBS, sections were stained using 3,3'-diaminobenzidine (DAB) tetrahydrochloride (Sigma-Aldrich, St. Louis, MO, USA) in the presence of 0.015% H_2_O_2_ in 0.05 M Tris–HCl buffer (pH 7.6). The reaction was stopped by washing in distilled water. Sections were counterstained with Haematoxylin-Mayer solution (Klinipath, Breda, The Netherlands), washed with tap water, dehydrated in alcohol and xylene and coverslipped in Pertex (VWR International, Radnor, PA, USA).

Double labeling was performed with ionized calcium-binding adapter molecule 1 (Iba-1; rabbit polyclonal, WAKO, Osaka Japan, 1:2000), glial fibrillary acidic protein (GFAP; mouse monoclonal, clone GA5, Sigma-Aldrich, St. Louis, MO, USA; 1:4000), neuronal nuclear protein (NeuN; mouse monoclonal, clone MAB377; Chemicon, Temecula, CA, USA; 1:2000) oligodendrocyte transcription factor 2 (Olig2; rabbit polyclonal, IBL International, Hamburg, Germany; 1:200) or albumin (rabbit polyclonal, DAKO, Glostrup, Denmark; 1:10,000). Sections were incubated overnight with primary antibody and the next day incubated with BrightVision poly-HRP anti-rabbit (Immunologic, Duiven, The Netherlands) for 30 min at room temperature and washed with PBS. Staining was developed using 3'-amino 9'-ethylcarbazole (AEC, Sigma-Aldrich, St. Louis, MO, USA) in 0.05 M Acetate buffer with 0.5% H_2_O_2_ filtered substrate solution. To remove the first primary antibody, sections were cooked in citrate buffer and then washed with PBS. Incubation with ferritin as second primary antibody was performed overnight followed by incubation with poly-alkaline phosphatase(AP)-anti-rabbit (Immunologic, Duiven, The Netherlands) for 30 min at room temperature the next day. AP activity was visualized with the AP substrate kit III Vector Blue (SK-5300, Vector Laboratories Inc., Burlingame, CA, USA). Sections incubated without the primary antibody were essentially blank. Finally, stained sections were air dried and coverslipped using VectaMount (H5000-60, Vector Laboratories Inc., Burlingame, CA, USA).

For DAB-enhanced Perl’s iron stain, the modified Meguro method was used [47]. After deparaffinization, tissue slides were incubated in freshly prepared, acidified 1% potassium ferrocyanide in distilled water (pH ~ 1.0 to 1.5) solution for 40 min at room temperature. Afterwards, slides were washed three times in distilled water and incubated in methanol containing 0.01 M NaN_3_ and 0.3% H_2_O_2_ for 75 min at room temperature. Slides were washed three times in 0.1 M PBS (pH 7.4) and then incubated in PBS containing 0.025% DAB and 0.005% H_2_O_2_ for 40 min at room temperature in the dark. For double labelling, slides were incubated with AEC instead of DAB, followed by primary antibody incubation overnight and AP-based color development as described in the preceding text. Color development was stopped by washing slides in distilled water. As negative control, one duplicate slide was incubated with non-acidified ferrocyanide solution (pH ~ 8.0) which did not show any staining. Additionally, coverslips with cells permeabilized with Triton X-100 prior to Perl’s stain as negative control did not show iron staining. Fetal astrocytes were counterstained with Safranin O (Merck-Millipore, Burlington, MA, USA). Finally, slides were dehydrated in ethanol and xylene and coverslipped using Pertex.

### Quantification of immunohistochemistry

Semi-quantitative analysis of surgically resected tissue was performed as described previously [2]. Briefly, tissue sections selected for investigation were evaluated by two independent observers for immunoreactivity (IR) using a scale 0–3 (0 = absent, 1 = weak, 2 = moderate, 3 = strong staining). All areas of the hippocampus (TLE-HS, SE) were examined and the score represents the predominant intensity found in each case. In addition, the number of cells positive for the investigated markers was evaluated (0 = absent, 1 = low, 2 = moderate, 3 = high). The product of intensity and number scores was taken to give the overall immunoreactivity score (IRS, Table 5). For iron/ferritin expression in astrocytes, cases were investigated for iron accumulation or ferritin expression in cell bodies of cells with astrocytic morphology.

**Table 5:** Immunohistochemical evaluation of 4-HNE, HO-1, ferritin and iron in post-mortem control vs. post-mortem SE and surgical TLE-HS brain tissue

	Control	SE	TLE-HS
Iron			
CA1	0 (0)	0 (0–3)	3 (3–6)**
DG	0 (0–3)	0 (0)	3 (3)*
Astrocytes	0 (0)	0 (0–2)	3 (0–3)*
Ferritin			
Astrocytes	0 (0)	3 (3–9)**	4.5 (3–6)**
4-HNE			
CA1	2 (0–6)	6 (6–9)*	9 (6–9)**
DG	0 (0–1)	1 (1–2)	3 (0–6)*
HO-1			
CA1	1.5 (0–3)	3 (3–6)*	9 (6–9)*
DG	1.5 (0–3)	3 (3–6)*	7.5 (3–9)*

The immunoreactivity score (IRS) is given as median with the range in brackets. Immunoreactivity was evaluated using a 0–3 scale (0 = absent, 1 = weak, 2 = moderate, 3 = strong staining). In addition, the number of positive cells was evaluated (0 = absent, 1 = low, 2 = moderate, 3 = high). The product of these two scores (IRS) was calculated for each case. *n* = 5 (control) *n* = 6, (TLE-HS), *n* = 5 (SE). *p < 0.05, **p < 0.01 using Mann–Whitney *U* test

Bold values indicate statistical significance

### Quantitative susceptibility mapping in epilepsy patients

Three patients (#1: male, 32 years old; #2: male, 29 years old; #3: male, 25 years old; Table 4) undergoing presurgical evaluation, including inpatient video monitoring, at the Department of Epileptology of the University Hospital Bonn were scanned interictally and postictally using a Siemens Magnetom Trio (3T) MRI-scanner. Seizure to postictal scan intervals for patients #1, #2, and #3 were 245 min, 166 min, and 72 min, respectively. Interictal scans were acquired at a minimum of 48 h after the last seizure and included a T1-weighted structural image (MP-RAGE, TE = 2.54 ms, TR = 1660 ms, flip angle = 9°, FOV = 171 × 279 × 206 mm, voxel size = 0.8 × 0.8 × 0.8 mm, TA = 6:32 min). For QSM, T2*-weighted MRI acquisitions were conducted using a gradient-recalled echo sequence with seven echo times (3D-FLASH, phase and magnitude image reconstruction, TE = 3.98/9.8/18.27/26.74/35.21/43.68/52.15 ms, TR = 59 ms, flip angle = 15° FOV = 172.5 × 230x144 mm, voxel size = 0.9 × 0.9 × 1.5 mm, TA = 10:34 min). Data processing included the application of the tool SEPIA [8], utilizing Laplacian-based phase unwrapping, Laplacian boundary value guided background field removal, and a final QSM modeling by thresholded K-space division. Susceptibility values were normalized by the whole-brain mean. Postictal and interictal images were linearly co-registered with the structural T1-weighted image and subtracted to quantify postictally increased magnetic susceptibility as surrogate marker for postictal iron accumulation. Masks of the presumable seizure-onset zone were manually demarcated according to the information gained in the multimodal presurgical evaluation. Using these masks, absolute susceptibility values were read out voxel-wise for both scans and Cohen’s d between the two distributions was assessed as metric of the effect size.

### RNA isolation for RNA sequencing

Total RNA for RNAseq, including the miRNA fraction, was isolated using the miRNeasy Mini kit (Qiagen Benelux, Venlo, The Netherlands) according to the manufacturer’s instructions. The concentration and purity of RNA were determined at 260/280 nm using a Nanodrop spectrophotometer (Ocean Optics, Dunedin, FL, USA) and RNA integrity was assessed using a Bioanalyzer 2100 (Agilent, Santa Clara, CA, USA).

### RNA-Seq library preparation and sequencing

All library preparation and sequencing were performed at GenomeScan (Leiden, The Netherlands). The NEBNext Ultra II Directional RNA Library Prep Kit for Illumina (New England Biolabs, Ipswich, MA, USA) was used for sample processing. Sample preparation was performed according to the protocol “NEBNext Ultra II Directional RNA Library prep Kit for Illumina” (NEB #E7760S/L). Briefly, mRNA was isolated from total RNA using oligo-dT magnetic beads. After fragmentation of mRNA, cDNA synthesis was performed. Next, sequencing adapters were ligated to the cDNA fragments followed by PCR amplification. Clustering and DNA sequencing was performed using the NovaSeq6000 (Illumina, Foster City, CA, USA) in accordance with manufacturers’ guidelines. All samples underwent paired-end sequencing of 150 nucleotides in length, the mean read depth per a sample was 47 million reads.

### Bioinformatics analysis of RNA-Seq data

The Bestus Bioinformaticus Decontamination Using Kmers (BBDuk) tool from the BBTools suite was used for adapter removal, quality trimming and removal of contaminant sequences (ribosomal/bacterial) [6]. A phred33 score of 20 was used to assess the quality of the read, any read shorter than 31 nucleotides in length was excluded from the down-stream analysis.

Reads were aligned directly to the human GRCh38 reference transcriptome (Gencode version 33) [28] using Salmon v0.11.3 [57]. Transcript counts were summarized to the gene level and scaled used library size and average transcript length using the R package tximport [78]. Genes detected in less than 20% of the samples in any diagnosis and with counts less than six across all samples were filtered out. The gene counts were than normalized using the weighted trimmed mean of M values (TMM) method using the R package edgeR [68]. The normalized counts were than log2 transformed using the voom function from the R package limma [67]. The subsequent differential expression was carried out using the R package limma. Differential expression testing was performed across all genes. Subsequently, a linear model was fit for each gene and moderated *t*-statistic was calculated after applying an empirical Bayes smoothing to the standard errors. Those genes with a Benjamini–Hochberg adjusted *p* value < 0.05 were considered differentially expressed. For correlation matrices, normalized counts of genes of interest were analyzed using Spearman’s rank correlation with a *p* value < 0.05 indicating significance.

To assess the similarities between the expression profiles of the manually curated gene sets from the reactome pathways [12] detoxification of reactive oxygen species (R-HSA-3299685) and glutathione conjugation (R-HSA-156590) combined as well as iron uptake and transport (R-HSA-917937) the Euclidean distance was calculated. These results were then visualized as heatmaps in R.

### Experimental animals

Experiments were performed on adult male Sprague Dawley rats (Envigo, Horst, The Netherlands) and were approved by the University Animal Welfare committee and performed in accordance with the guidelines of the European Community Council Directives 2010/63/EU. The rats were housed individually in a controlled environment (21 ± 1 °C; humidity 60%; lights on 08:00 a.m.–8:00 p.m.; food and water available ad libitum). Rat brain tissue utilized in this study was retrieved from archived brain tissue from the electrically induced rat post-SE model prepared as described previously [5].

For immunohistochemistry on paraffin-embedded rat tissue against ferritin, iron and HO-1, rats were deeply anesthetized with pentobarbital (Euthasol, AST Farma, Oudewater, The Netherlands, 60 mg/kg i.p.) and perfused via the ascending aorta (300 mL 0.37% Na_2_S followed by 300 mL 4% PFA in 0.1 M phosphate buffer, pH 7.4). Rats were perfused at three different time points after SE, each corresponding to the early and late stages of epileptogenesis: the acute phase (1 day post‐SE, *n* = 5) and the chronic phase (7 months post‐SE, when recurrent spontaneous electrographic seizures are evident, *n* = 5; [25]). Control rats (*n* = 5) that were implanted with EEG electrodes, but not stimulated, were also included. The brains were post‐fixated overnight, dissected and embedded in paraffin. Tissue was sectioned sagittally at 6 μm and mounted on pre‐coated glass slides (Star Frost, Waldemar Knittel, Braunschweig, Germany).

For quantitative real-time PCR analysis, protein and iron analysis, a separate cohort of rats were decapitated 1 day after SE (acute phase), 1 week after SE (latent phase) or 3.5 months after SE (chronic phase). Electrode‐implanted control rats were also included. The brain was dissected and the parahippocampal cortex [PHC; protein analysis *n* = 5 (control, chronic), *n* = 6 (acute); RNA *n* = 5 (control, acute, chronic), *n* = 6 (latent)], which includes mainly the entorhinal cortex (EC) and parts of the peri-rhinal and posterior piriform cortex, was removed by incision at the ventro‐caudal part underneath the rhinal fissure until approximately 5 mm posterior to bregma. Additionally, the whole hippocampus was removed as well and used for iron assays [*n* = 3 (acute), *n* = 4 (control), *n* = 6 (chronic)] or dissected into CA1 [*n* = 5 (control, latent, chronic), *n* = 6 (acute) and dentate gyrus (DG; *n* = 4 (control, latent), *n* = 5 (acute, chronic)] for RNA analysis. Animals sacrificed during the chronic phase and used for protein and iron determination had daily SRS. All material was frozen on dry ice and stored at − 80 °C until use.

### Mouse slice preparation and analysis

Mouse brain slices were prepared from wild-type C57/BL6 mice ranging from 4 to 6 weeks of age. Mouse rather than rat brain slices were chosen due to their improved experimental utility in this experimental set-up as described elsewhere [51]. The protocol was approved by the University Animal Welfare committee and performed in accordance with the guidelines of the European Community Council Directives 2010/63/EU. Mice were decapitated, the brain was dissected within 1 min and placed in ice-cold slicing artificial cerebrospinal fluid (ACSF; 208 mM sucrose, 2 mM KCl, 0.5 mM CaCl_2_, 2 mM MgSO_4_, 5 mM MgCl_2_, 1.25 mM KH_2_PO_4_, 10 mM d-glucose, 26 mM NaHCO_3_, 3 mM pyruvate, 1 mM l-ascorbic acid pH 7.4) bubbled with carbogen (95% O_2_/5% CO_2_). Horizontal hippocampal brain slices were cut at a 10° angle using a vibratome (Leica VT1200S, Leica, Germany) at 400 μm (for recording and assay lysates) or 100 μm (for stainings) and submerged in ice-cold slicing ACSF, bubbled with carbogen. For 4-aminopyridine (4-AP) recordings, partially disconnected slices were prepared to restrain fast CA3 driven interictal-like activity spreading to the parahippocampal cortices by cutting the Shaffer collaterals with a micro-blade as described previously [52]. Thereafter, slices were washed and allowed to recover in holding ACSF (115 mM NaCl, 2 mM KCl, 1.25 mM KH_2_PO_4_, 1.3 mM MgSO_4_, 2 mM CaCl_2_, 25 mM d-glucose, 26 mM NaHCO_3_, 1 mM l-ascorbic acid pH 7.4 in distilled water at 300 mOsm/kg), bubbled with carbogen for 45–60 min at room temperature.

For multi-electrode array (MEA) recordings, brain slices were prewarmed in holding ACSF at 30–32 °C for 30 min. For 4-AP recordings, slices were additionally pre-incubated another 30 min in holding ACSF with 250 μM 4-AP. For recordings, slices were transferred to the recording set-up and placed in the recording chamber, held down via a custom-made platinum anchor. The recording chamber was perfused with prewarmed recording ACSF (115 mM NaCl, 2 mM KCl, 1.25 mM KH_2_PO_4_, 1 mM MgSO_4_, 2 mM CaCl_2_, 25 mM d-glucose, 26 mM NaHCO_3_, 1 mM l-ascorbic acid pH 7.4 in distilled H_2_O at 300 mOsm/kg) via a PH01 heating cannula (Multichannel systems, MCS, Reutlingen, Germany) at 37 °C before entering the 32 °C MEA bath, creating a fluid temperature in the bath of roughly 32–34 °C. Slices were allowed to equilibrate for 10 min and subsequently recorded for 1–2 h. The MEA consisted of 60 TiN/SiN planar electrodes, including the reference electrode, with 200 μm between the center of each electrode and was placed in a MEA mini 1200 system head-stage (Multichannel systems, Reutlingen, Germany). Signals were recorded at 10 kHz, with a 200 Hz low-pass filter using the Multichannel experimenter program (v 2.17.2) and analyzed using the Multichannel analyzer program (v 2.17.2) (Multichannel systems, Reutlingen, Germany). For analysis of spike frequency upon 4-AP stimulation, automated spike counting with a cut-off of five standard deviations in a 30 min time-window of stable recording was applied. Spikes from 2 to 3 channels per area (CA3, EC) were quantified and averaged to yield the average frequency per minute per brain area.

Slices that were not used for MEA recordings were incubated in recording ACSF with 250 μM 4-AP, 1 mM monosodium l-glutamate, 200 μM FAC, 10 μM Hemin or a combination and optionally 10 µM nifedipine or 20 µM MK801 (Merck-Millipore, Burlington, MA, USA) for 1 h at 37 °C. Afterwards, slices were washed in distilled water and either lysed in 5 M NaCl with 1% Tween-20 (iron assay), Qiazol (RNA analysis) or fixed for 24 h in 4% PFA in 0.1 M phosphate buffer, pH 7.4 (stainings). If not stated otherwise, all chemicals were purchased from Sigma-Aldrich (Merck-Millipore, Burlington, MA, USA).

### Cell culture and stimulation

Primary fetal astrocyte-enriched cell cultures were obtained from human fetal brain tissue (cortex, 14–19 gestational weeks) from medically induced abortions. All material was collected from donors from whom written informed consent for the use of the material for research purposes was obtained by the Bloemenhove kliniek (Heemstede, The Netherlands). Tissue was obtained in accordance with the Declaration of Helsinki and the Amsterdam UMC Research Code provided by the Medical Ethics Committee. Cell isolation was performed as described previously [83]. Briefly, large blood vessels were removed, after which the tissue was mechanically minced into smaller fragments and enzymatically digested by incubating at 37 °C for 30 min with 2.5% trypsin (Sigma-Aldrich, St. Louis, MO, USA). Tissue was washed with incubation medium containing Dulbecco’s modified Eagle’s medium (DMEM)/HAM F10 (1:1) medium (Thermo Fisher Scientific, Waltham, MA, USA), supplemented with 100 units/mL penicillin, 100 µg/mL streptomycin, 1% glutamine (Thermo Fisher Scientific, Waltham, MA, USA) and 10% fetal calf serum (FCS; Thermo Fisher Scientific, Waltham, MA, USA) and triturated by passing through a 70 µm mesh filter. Cell suspension was incubated at 37 °C, 5% CO_2_ for 48 h to let glial cells adhere to the culture flask before it was thoroughly washed with PBS to remove excess myelin and cell debris. Cultures were subsequently refreshed twice a week. Cultures reached confluence after 2–3 weeks. SHSY5Y neuroblastoma cells (ATCC CRL-2266™; gift from Prof. M. Perluigi) were cultured in DMEM/F-12 (Thermo Fisher Scientific, Waltham, MA, USA) with 5% Glutamine, 100 units/mL penicillin, 100 µg/mL streptomycin, 1% glutamine and 10% FCS.

Cell cultures for experiments were obtained by trypsinizing and sub-plating cells onto 96-well (1 × 10^4^ cells/well for MTT assay) and poly-L-lysine (PLL, 15 µg/mL, Sigma-Aldrich, St. Louis, MO, USA)-pre-coated 12- and 24-well plates (Greiner Bio-One, Kremsmünster, Austria; 5 × 10^4^ cells/well for RNA isolation and PCR, 5 × 10^4^ cells/well with coverslips for immunocytochemistry). Fetal astrocyte cultures were used at passage 2–5 for all experiments and SHSY5Y cells at passage ≥ 13. Stimulation was done either with chronic OS (1 mU (SHSY5Y)/2.5 mU (astrocytes) glucose oxidase (GO) for 72 h with exchange of stimulation medium every 24 h to ensure sufficient glucose concentration), acute OS (100 μM (SHSY5Y)/500 μM (astrocytes) H_2_O_2_ for 3 h), iron (200 μM ferric ammonium citrate (FAC) for 24 h), hemin (1 μM (SHSY5Y)/10 μM (astrocytes) for 24 h) or a combination at 37 °C, 5% CO_2_. For albumin stimulation, human fetal astrocytes were sub-plated in PLL-coated 12-well plates and serum-starved by incubation in DMEM/HAM F10 (1:1) medium with 100 units/mL penicillin, 100 µg/mL streptomycin, 1% glutamine supplemented with G5 (Thermo Fisher Scientific, Waltham, MA, USA) for 48 h. Thereafter, fetal astrocytes were exposed to 300 µM bovine serum albumin (BSA; Roche Applied Science, Basel, Switzerland) or 300 µM recombinant human serum albumin (HSA; Sigma-Aldrich, St Louis, MO, USA) for 24 h or 48 h. After stimulation cells were washed with PBS and lysed in 700 μL Qiazol for RNA analysis or washed with PBS, fixed for 15 min in 4% PFA at room temperature and washed again with PBS for immunocytochemistry. For high-mobility group box 1 (HMGB-1) and interleukin 6 (IL-6) analysis, cell supernatant was collected, centrifuged at 12,000×*g* for 5 min at 4 °C and kept at -80 °C until analysis.

### MTT viability assay

Cell viability was determined by the 3-(4,5-dimethylthiazol-2-yl)-2,5-diphenyl tetrazolium bromide (MTT; Sigma-Aldrich, St Louis, MO, USA) cell viability assay. Subsequent to stimulation, 0.5 mg/ml MTT in complete medium was added to each well and the plate was incubated for 1 h at 37 °C in 5% CO_2_. The reaction mixture was aspirated and 100 μL lysis buffer (4 mM HCl, 0.1% Nonidet P-40 in isopropanol) was added to each well to stop color development and disrupt cells. Plates were agitated to ensure complete lysis of MTT crystals and cell viability was determined by measuring optical density at 570 nm wavelength using a microplate reader (BMG Labtech, Ortenberg, Germany). Absorbance of treated cells was plotted relative to control cells.

### Human astrocyte and SHSY5Y cell culture supernatant immunoassays

For analysis of HMGB-1 (IBL International, Hamburg, Germany) or IL-6 (Sanquin, Amsterdam, The Netherlands) concentration in cell culture supernatant, procedures were performed according to the manufacturer’s instructions. Absorbance of samples was read at 450 nm using a microplate reader and concentration was determined using a HMGB-1/IL-6 standard curve.

### Electron microscopy

SHSY5Y neuroblastoma cell cultures were fixed after stimulation using 2% paraformaldehyde (PFA) and 0.2% glutaraldehyde in 0.4 M PHEM buffer [240 mM piperazine-*N*,*N*′-bis(2-ethanesulfonic acid) (PIPES), 100 mM 4-(2-hydroxyethyl)-1-piperazineethanesulfonic acid (HEPES), 8 mM MgCl_2_, 40 mM ethylene glycol-bis(β-aminoethyl ether)-*N*,*N*,*N*′,*N*′-tetraacetic acid (EGTA)] for 24 h. Subsequently, cells were washed twice with 0.1 M PBS containing 0.15 M glycine and stained with 1% osmium tetroxide for 1 h. Thereafter, cells were dehydrated in a series of 50, 70, 80, 96 and 100% ethanol. Then, cells were carefully detached from plastic wells and placed in 50% epoxy resin for 1 h at room temperature, then 100% resin at 65 °C overnight. Samples were polymerized and sectioned in ultrathin sections (70 nm) which were placed on copper grids and visualized with a FEI Tecnai T12 Transmission Electron Microscope and G2 Spirit Biotwin using Veleta and Xarosa camera plus integrated Radius software.

### RNA isolation and quantitative real-time PCR

For RNA isolation, human, rat, mouse and cell culture material was homogenized in 700 µL Qiazol Lysis Reagent (Qiagen Benelux, Venlo, The Netherlands). Total RNA was isolated using phenol/chloroform extraction. To this end, 140 μL chloroform was mixed with Qiazol lysate and centrifuged at 12,000×*g* for 15 min at 4 °C. The aqueous phase was collected and mixed 1:1 with ice-cold isopropanol and 1 μL of glycogen blue (GlycoBlue, Thermo Fisher Scientific, Waltham, MA, USA) and incubated at − 20 °C overnight followed by centrifugation at 20,000×*g* for 35 min at 4 °C. RNA pellets were washed two times with ice-cold 80% ethanol, air-dried and dissolved in RNase-free water.

Concentration and purity of RNA were determined at 260/280 nm using a Nanodrop 2000 spectrophotometer (Thermo Fisher Scientific, Waltham, MA, USA). To evaluate mRNA expression, 250 ng of cell culture derived total RNA or 500 ng tissue-derived total RNA were reverse-transcribed into cDNA using oligo-dT primers. PCRs were run on a Roche Lightcycler 480 thermocycler (Roche Applied Science, Basel, Switzerland) using the reference genes chromosome 1 open reading frame 43 (*C1ORF43*) and elongation factor 1α (*EF1-α*) for human, Cyclin A (*CycA*) and glyceraldehyde 3-phosphate dehydrogenase (*GAPDH*) for rat and TATA box-binding protein (*Tbp*) and hypoxanthine phosphoribosyltransferase 1 (*Hrpt-1*) for mouse mRNA (see Online resource 8 for primer sequences). The PCR mix contained 1 μL cDNA, 2.5 μL SensiFAST SYBR Green NoROX kit (Bioline Reagents Limited, London, UK), 0.4 μM of forward/reverse primers plus water to a final volume of 5 μL/well. PCR reactions were run in duplicates and a negative control containing water instead of cDNA was included for each gene in each run. Cycling conditions were as follows: initial denaturation at 95 °C for 5 min, followed by 45 cycles of denaturation at 95 °C for 15 s, annealing at 65 °C for 5 s and extension at 72 °C for 10 s. Fluorescence of the sample was measured via single acquisition mode at 72 °C after each cycle. Quantification of data was performed using LinRegPCR as described elsewhere [83].

### Western blot analysis

For protein analysis, rat PHC or 40–50 mg of frozen human hippocampal tissue was mixed with lysis buffer containing 10 mM Tris (pH 8.0), 150 mM NaCl, 10% glycerol, 1% NP-40, 0.4 mg/ml sodium orthovanadate, 5 mM EDTA (pH 8.0), 5 mM NaF, and protease inhibitors (Protease inhibitor cocktail tablets, Roche Diagnostics, Mannheim, Germany) and homogenized by pottering and subsequent trituration. Homogenates were centrifuged at 12,000×*g* for 10 min at 4 °C and the supernatant was used for further analysis. Protein concentrations were estimated using the bicinchoninic acid assay (BCA) (Sigma-Aldrich, St. Louis, MO, USA).

For western blotting, rat/human protein extract was boiled at 100 °C for 8 min. Equal amounts of protein lysate (15 μg/lane) were then separated using sodium dodecyl sulfate polyacrylamide gel electrophoresis on a gradient Bolt 4–12% Bis–Tris gel (Thermo Fisher Scientific, Waltham, MA, USA) and electrotransferred for 90 min at 100 V to polyvinylidene difluoride (PVDF) membranes (Immobilon-P; Merck, Darmstadt, Germany). Blots were then blocked with 5% skim milk in Tris-buffered saline with 0.1% Tween20 (TBS-T; 20 mM Tris, 150 mM NaCl, 0.1% Tween20, pH 7.5) for 1 h at room temperature. Membranes were cut and incubated with primary antibodies overnight at 4 °C in 5% skim milk in TBS-T and subsequently washed 3 × 10 min in TBS-T. This was followed by incubation with horseradish peroxidase-coupled secondary antibodies for 1 h at room temperature in 5% skim milk in TBS-T. After three washes, the membranes were incubated with ECL (PLUS) Western blotting detection reagent (GE Healthcare Europe, Diegen, Belgium). Blots were digitized using an ImageQuant LAS 4000 system (GE Healthcare Europe, Eindhoven, The Netherlands). β-tubulin was used as loading control. Precision Plus Protein Dual Color Standard (Bio-Rad, Richmond, CA, USA) was used to determine the molecular weight of proteins. Blots were probed with the same antibodies as for IHC unless otherwise specified: ferritin (1:1000), HO-1 (1:1000), 4-HNE (1:1000), FPN-1 (SLC40A1; rabbit polyclonal, Novus Biologicals, Abingdon, UK; 1:1000), glutamate cysteine ligase catalytic subunit (γGCSc referred to as GCLC; polyclonal rabbit, Santa Cruz Biotechnology, Dallas, TX, USA; 1:1000), β-tubulin (mouse monoclonal, clone D66, Sigma-Aldrich, St. Louis, MO, USA; 1:30,000), anti-mouse/HRP (SouthernBiotech, Birmingham, AL, USA; 1:2500) or anti-rabbit/HRP (Agilent Technologies, Middelburg, The Netherlands; 1:2500). To quantify the blots, band intensities of individual proteins were measured densitometrically using ImageJ (v. 1.51; U.S. National Institute of Health, Bethesda, MD, USA) and normalized to β-tubulin.

### Iron determination in tissue lysates

For determination of total cell culture or tissue iron, lysates in protein lysis buffer were mixed 1:10 with assay working buffer (0.2 M l-ascorbic acid (Sigma-Aldrich, St. Louis, MO, USA) in 0.4 M acetate buffer, pH 4–4.5) containing 25 mM 3-(2-Pyridyl)-5,6-di(2-furyl)-1,2,4-triazine-5′,5′′-disulfonic acid disodium (Ferene-S; Merck, Darmstadt, Germany). Samples were mixed and incubated ≥ 20 h at room temperature. Thereafter, samples were mixed and centrifuged at 20,000×*g* for 5 min and 50 μL supernatant was transferred in duplicates to a 96-well flat-bottom plate (Greiner Bio-One, Kremsmünster, Austria) and measured at 595 nm in a microplate reader. For quantification of total iron, FAC standards (0–50 μg iron) were used (1 M FAC corresponding to ~ 45.846 mg iron). Iron concentrations were plotted relative to protein input as determined by the BCA method.

### Statistical analysis

Statistical analysis was performed with GraphPad Prism software version 5.01 (Graphpad software Inc., La Jolla, CA, USA) using a non-parametric Mann–Whitney *U* test or Kruskal–Wallis test followed by Dunn *post hoc* or Two-way ANOVA followed by Bonferroni correction for analysis for multiple groups. *p* value < 0.05 was assumed to indicate a significant difference. Data are displayed as bar graphs with standard deviation (SD) or Tukey style box plot with interquartile range from 25 to 75th percentile plus outliers. Correlation analysis was performed using Spearman’s rank correlation.

## Results

### Neuronal and astrocytic iron accumulation in the hippocampus of SE or TLE-HS patients are accompanied by changes in iron metabolism

**Fig. 1:**
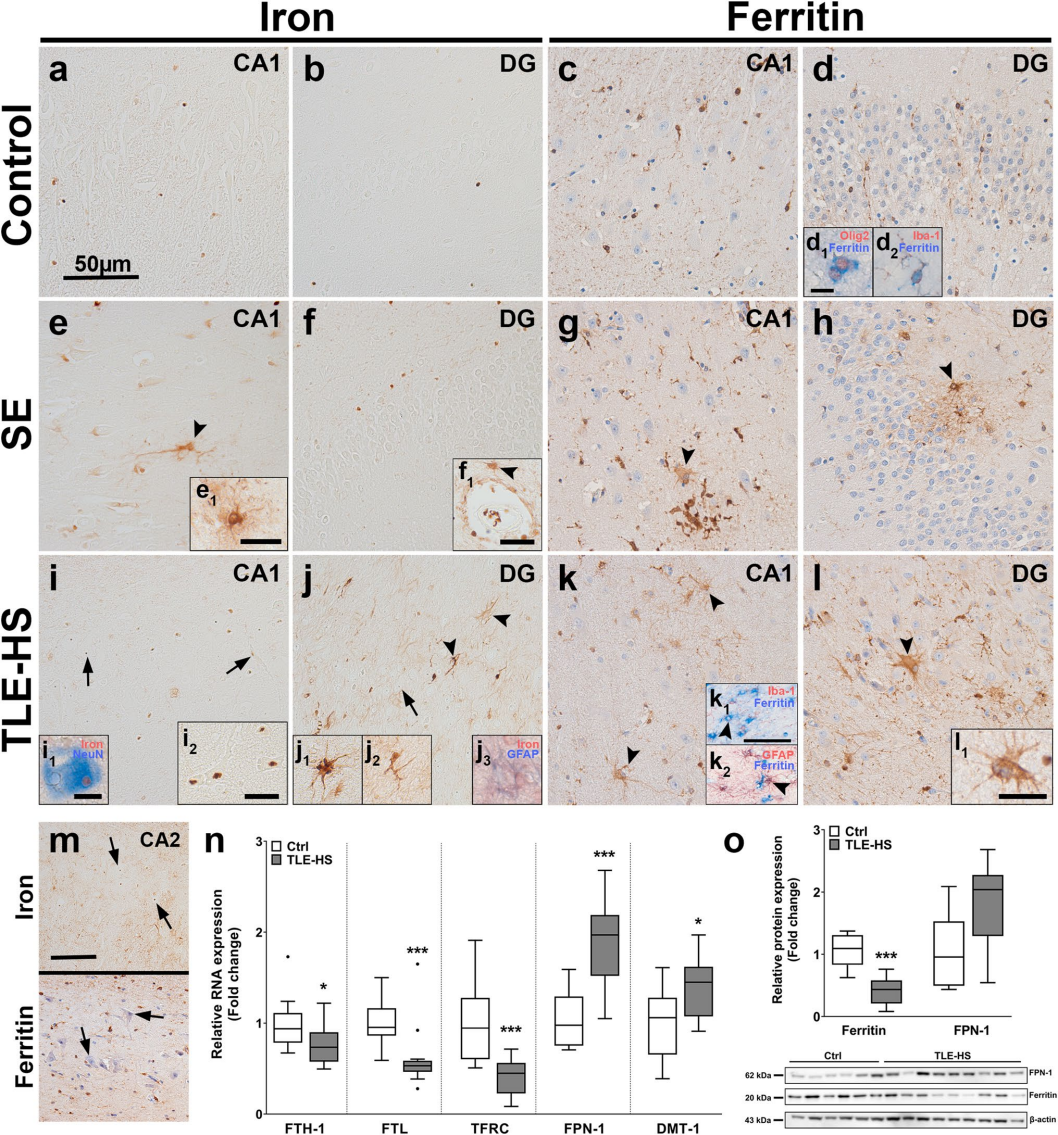
SE and TLE-HS hippocampal tissue is characterized by iron accumulation in pyknotic neurons, glial iron and ferritin accumulation and altered iron metabolism. **a**–**d** Iron could be sparsely detected, predominantly in white matter inside oligodendrocytes, while ferritin protein expression in autopsy control hippocampus could be detected exclusively in oligodendrocytes (d_1_) and microglia (d_2_) in CA1 and DG. **e**, **f** In SE tissue, iron accumulation could be detected perivascularly (f_1_) and in cells with glial morphology (e, e_1_, arrowhead). **g**, **h** Ferritin was primarily detected in microglia but also cells with astrocytic morphology (arrowheads). **i**, **j** Iron accumulation in TLE-HS hippocampi was detected in neuronal nuclei and specifically nucleoli in CA1 (arrows) and hilar neurons (i_1, 2_), especially in pyknotic neurons. Moreover, iron accumulation could also be found in cells with astrocytic (j_1_) and microglial (j_2_) morphology (arrowheads). Double labeling revealed co-localization of GFAP and iron in some astrocytes (j_3_). **k**, **l** Surgically resected hippocampi from TLE-HS patients displayed ferritin expression in microglia, but also cells with astrocyte morphology (l_1_, arrowheads). Double labelling revealed GFAP-positive Iba-1-negative cells that expressed ferritin (k_1, 2_, arrowheads). **m** In the CA2 subfield with preserved neurons, iron was detected in glial processes and neuronal nucleoli (arrows), while ferritin localized to microglia. **n** FTH-1 RNA expression did not differ from autopsy control in TLE-HS hippocampal tissue, while FTL and TFRC were reduced. Moreover, FPN-1 and DMT-1 RNA expression were elevated. **o** Downregulation of ferritin was also confirmed via Western blot, while FPN-1 was not different. Sections **c**, **d**, **g**, **h**, **k**, **l** were counterstained with hematoxylin. Scale bars: 50 µm in a (representative for **a**–**l**), m and k_1_ (representative for k_2_), 15 µm in inserts e_1_, f_1,_ i_2,_ l_1_; 10 µm in i_1_ (representative for j_1-3_), 5 µm in d_1,2_; arrows = neurons, arrowheads = glia. **n**, **o** Mann–Whitney *U* test. Data are expressed relative to expression observed in autopsy controls and displayed as Tukey box plots; **p < 0.01, ***p < 0.001. **n**: *n* = 10 autopsy control vs. *n* = 13 TLE-HS; **o﻿**: *n* = 6 autopsy control vs. *n* = 9 TLE-HS

To determine iron content in the hippocampus of patients with SE or TLE, the distribution of tissue iron as well as the expression of the primary iron storage protein ferritin were assessed (see also Online resource 1a-f for lower magnification overview). In the hippocampus of control tissues (Fig. 1a, b), iron was mainly present in white matter and cells with oligodendroglial morphology (Online resource 1a, m). Since formalin fixation might induce changes in iron ionization [27] and promote leaching of iron from tissue upon fixation [11, 73] iron staining between frozen and paraffin-embedded tissue was compared. While the overall staining intensity for iron was slightly higher in fresh frozen tissue, the iron localization was preserved in formalin-fixed paraffin-embedded tissue (Online resource 1n), indicating that these tissues are suitable for analyzing tissue iron. SE tissue had a higher iron load (Fig. 1e, f) as compared to control tissue, with occasional iron accumulation in glial processes (Fig. 1e, arrowhead) and cells with astrocyte morphology (Fig. 1e_1﻿_). Additionally, punctate iron staining could be detected perivascularly and occasional iron accumulation was observed in perivascular glia (Fig. 11f_1﻿_, arrowhead). Iron in TLE-HS tissue was detected in the nucleus and even stronger in the nucleolus of CA1 and hilar neurons, especially with pyknotic morphology (Fig. 1i, i_1,2﻿_, j, arrows). Iron accumulation could also be detected in microglia and astrocytes (Fig. 1j arrowheads, j_1–3_) as well as the neuropil. Iron also accumulated in glia around preserved neurons in the CA2 area as well as in neuronal nuclei (Fig. 1m).

Ferritin expression in hippocampi of autopsy control tissue was detected in oligodendrocytes and microglia (Fig. 1d_1﻿_, d_﻿2_). Ferritin-positive neurons could not be detected in the CA1 or the DG, while fine ferritin-positive astrocytic-like processes could be detected in the hippocampus and EC (Fig. 1c, d; Online resource 1o). In all SE cases, ferritin was detected in the hippocampus in microglia and astrocytes (Fig. 1g, h arrowheads), as well as in the EC (Online resource 1o). Ferritin expression in the hippocampus of TLE-HS cases could be detected predominantly in microglia and more frequently in astrocytes compared to control and SE tissue (Fig. 1k, l, l_1_). Though not all TLE-HS hippocampi contained adjacent EC and, thus, could not be quantified, those cases with EC displayed a similar high expression of ferritin in astrocytes (Online resource 1o). Double labeling revealed ferritin expression in astrocytes and microglia (Fig. 1k_1﻿_, k_2_, arrowheads). Ferritin expression around preserved neurons in the CA2 was primarily found in microglia (Fig. 1m). A summary of the immunohistochemical evaluation for SE and TLE-HS tissue compared to autopsy control can be found in Table 5. Quantification of gene expression revealed alterations in iron metabolism in TLE-HS brain tissue, with lower RNA expression of ferritin heavy (FTH-1) and light chain (FTL) (Fig. 1n). On the protein level, ferritin expression was markedly lower as compared to control (Fig. 1o). These results indicate increased iron deposition and marked changes in iron metabolism in SE and TLE-HS tissue.

Expression of iron transporters was also altered in TLE-HS; RNA expression was lower for FPN-1 and DMT-1, while TFRC showed higher expression (Fig. 1n), although FPN-1 protein levels were not significantly higher (Fig. 1o). TFRC in control cases was found mostly in endothelium of vessels and DG neurons (Online resource 2a, a_1_, d). In SE cases, TFRC could be found in some neuronal inclusions in the CA1 as well as in astrocytes, while TLE-HS tissue revealed overall weak neuronal expression with occasional strong expression in some neurons and astrocytes (Online resource 2b, c, e, f). DMT-1 displayed perinuclear staining as well as reactivity in intracellular inclusions and cell membranes in neurons in control tissue (Online resource 2 g, j). In SE tissue, DMT-1 expression was stronger perinuclear in neurons compared to controls (Online resource 2 h, k). In TLE-HS tissue, a markedly higher DMT-1 expression could be found as compared to controls, primarily in some CA1 neurons, but predominantly in astrocytes (Online resource 2i, l).

To determine if iron accumulation is associated with BBB leakage in TLE-HS hippocampi, iron and albumin co-labeling was performed. Perivascular iron accumulation in glial processes could be detected and co-localized with albumin in cells with astrocyte morphology (Online resource 2 m, o, p arrowheads). In addition, albumin could be found in neurons which also co-localized with iron (Online resource 2m_1_,_2_, p, arrows). However, there were also areas of strong extracellular albumin reactivity with nuclear iron accumulation in neurons as well as areas with iron but no albumin (Online resource 2n, p).

In addition to SE and TLE-HS brain tissue, autopsy tissue from patients who died following TBI and stroke were investigated in a small cohort of patients with varying post-injury delays before death. Though these tissues were retrieved from cortical lesions, the general response of cells to the injury and consequent iron loading of the tissue serving as reference were of interest. Blood extravasation acutely after trauma (< 48 h) was followed (> 48 h) by iron accumulation in some neurons, but predominantly perivascular cells with microglial and macrophage morphology (Online resource 3a, b). This was mirrored by strong perivascular ferritin expression in morphologically similar cells (Online resource 3d, e). Lesions from patients who developed epilepsy after TBI (> 6 months) contained areas of high iron reactivity in the neuropil and glia around vessels as well as neuronal iron accumulation in cytoplasm and nucleolus (Online resource 3c). Ferritin expression could be detected primarily in microglia, whereas ferritin expression in cells with astrocyte morphology was apparent in older lesions (Online resource 3f, f_1_). Similar to TBI tissue, acute stroke lesions (2–4 days) displayed perivascular iron related to bleeding with little ferritin expression (Online resource 3 g, j). Older lesions (5–6 days) displayed iron accumulation and ferritin expression in glia and macrophages, as well as cytoplasmic iron accumulation in neurons (Online resource 3 h, k). Even older lesions (2 weeks) showed iron mostly contained in cells with glial and macrophage morphology and ferritin-expressing glia and macrophages around vessels, some shaped like astrocytes (Online resource 3i, l).

### Neuronal oxidative damage and oxidative stress response are elevated in the hippocampus of patients with SE or TLE-HS

**Fig. 2:**
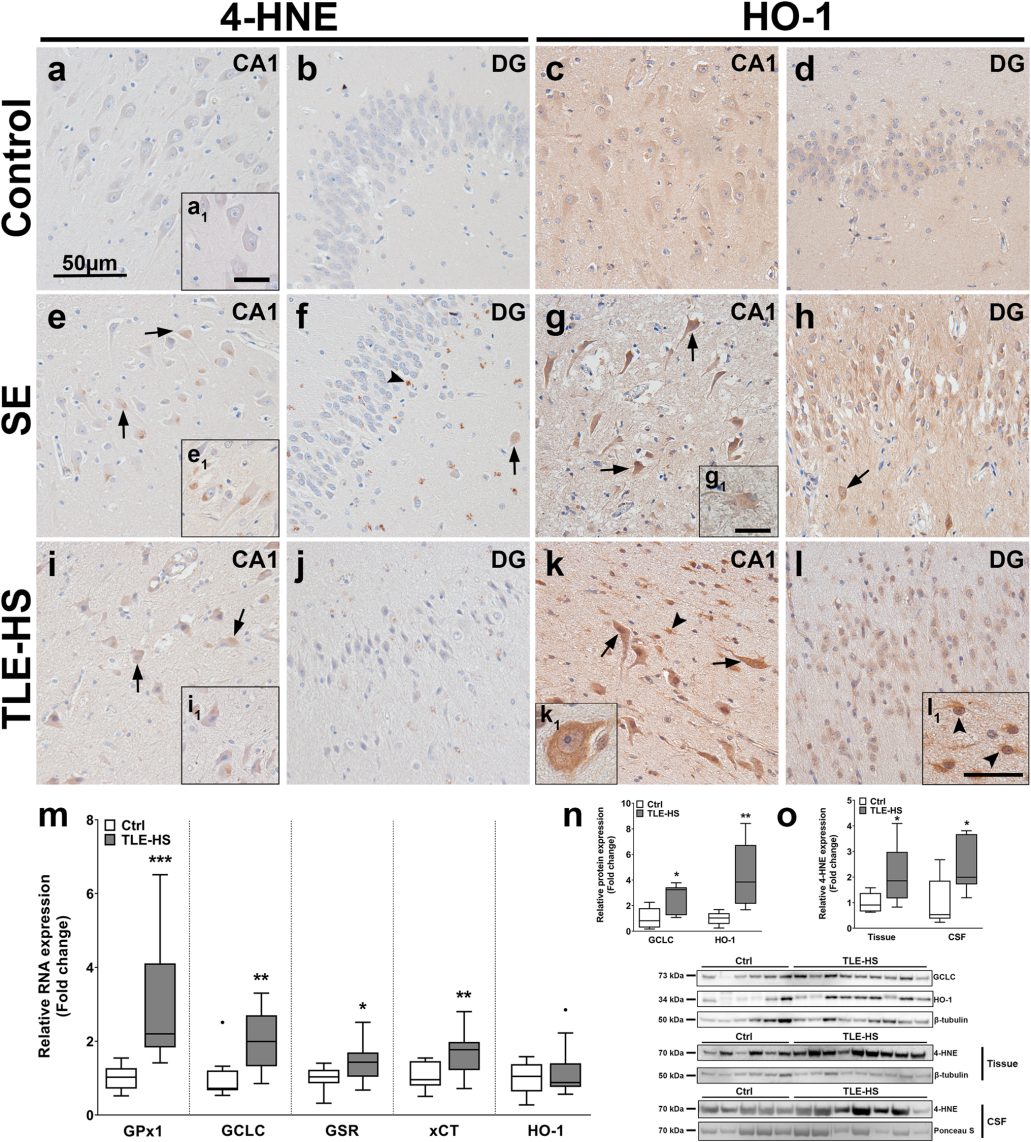
SE and TLE-HS hippocampus tissue display higher reactivity for the oxidative damage marker 4-HNE, higher expression of glutathione synthesis enzymes and the oxidative stress marker HO-1. **a**, **b** Expression of 4-HNE in the hippocampus of autopsy control hippocampi was restricted primarily to CA1 neurons (a_1_) displaying very low reactivity in intracellular deposits. **c**, **d** Similarly, very low expression of HO-1 could be detected in CA1 neurons but not the DG. **e**, **f** In the hippocampus of SE patients, neurons in CA1 (e_1_) and DG displayed more reactive intracellular deposits (arrows). Additionally, some glial cells revealed intracellular deposits in the DG (arrowhead). **g**, **h** HO-1 in SE tissue was strongly expressed in CA1, DG as well as hilar interneurons (h, arrow). In addition, cells with astrocyte morphology showed high HO-1 expression (g_1_). **i**, **j** The hippocampus of surgically resected TLE-HS brain tissue revealed 4-HNE deposits in CA1 neurons (i_1_) and DG glia (arrows). **k**, **l** HO-1 expression in TLE-HS brain tissue was markedly higher, primarily in CA1 and DG neurons (arrows), but also hilar interneurons (k_1_) and cells with glial morphology (l_1_, arrowheads)**. m** RNA quantification of TLE-HS hippocampi showed higher expression of glutathione metabolic genes GPx1, GCLC, GSR and xCT, while HO-1 RNA was not different from autopsy control. **n** Total protein quantification of TLE-HS hippocampi revealed higher GCLC and HO-1 expression compared to autopsy control. **o** Additionally, 4-HNE expression was higher in tissue and cerebrospinal fluid (CSF) of TLE-HS patients compared to control. Sections **a**–**l** were counterstained with hematoxylin. Scale bars: 50 µm in a (representative for **a**–**l**), 20 µm in inserts a_1_ (representative for b_1_, c_1_) and l_1_, 20 µm in g_1_ (representative for k_1_); arrows = neurons, arrowheads = glia. **m**, **n** Mann–Whitney *U* test. Data are expressed relative to expression observed in autopsy controls and displayed as Tukey box plots; *p < 0.05, **p < 0.01, ***p < 0.001. **m**: *n* = 10 autopsy control vs. *n* = 13 TLE-HS; **n**: *n* = 6 autopsy/CSF control vs. *n* = 9 TLE-HS; **o**: *n* = 6 autopsy/CSF control vs. *n* = 9 TLE-HS

Since OS and iron metabolism are intimately related, we next investigated which cell types are affected most by OS in SE and TLE-HS brain tissue by examining 4-HNE and HO-1 expression (respective markers of lipid peroxidation and ROS response; a low magnification overview can be found in Online resource 1 g–l). For 4-HNE, low-to-no expression was detected in nuclei of CA1 neurons, while the DG was almost blank in control hippocampus (Fig. 2a, a_1_, b). HO-1 expression in CA1 and DG neurons of control tissue displayed low, but detectable expression (Fig. 2c, d). In SE tissue, we found many 4-HNE-positive perinuclear intracellular deposits, predominantly in CA1 neurons and occasionally also in interneurons and glial cells in the DG (Fig. 2e, e_1_, f). HO-1 expression in SE tissue was markedly higher in CA1 and DG as compared to controls, especially in pyknotic neurons and hilar interneurons (Fig. 2g, h, arrows). In addition, HO-1 expression could also be detected in astrocytes (Fig. 2g_﻿1_). In TLE-HS tissue, not only 4-HNE reactive deposits, similar to SE, but also cell membrane reactivity was found mostly in CA1 neurons, especially within pyknotic neurons (Fig. 2i, i_1_). DG neurons also displayed higher 4-HNE reactivity but much less than CA1 neurons (Fig. 2j). Similar to SE tissue, HO-1 expression in TLE-HS was very high in CA1 and DG neurons, as well as hilar interneurons (Fig. 2k, k_1_, l). Additionally, glial cells also displayed higher HO-1 expression in some cases of TLE-HS (Fig. 2l_1﻿_). A summary of the immunohistochemical evaluation for SE and TLE-HS tissue compared to autopsy control can be found in Table 5. Quantification of gene expression revealed a marked increase in glutathione metabolism indicated by higher RNA expression of glutathione peroxidase 1 (GPx1), GCLC, glutathione disulfide reductase (GSR) and the glutamate/cysteine antiporter system Xc^−^ light subunit (xCT [SLC7A11]) in TLE-HS as compared to control, while HO-1 expression remained unchanged (Fig. 2m). Higher expression of not only GCLC but also HO-1 was detected upon investigation of total protein (Fig. 2n). This was accompanied by a higher 4-HNE reactivity of the most prominent protein band at 70 kDa in TLE-HS tissue and cerebrospinal fluid (CSF) compared to control (Fig. 2o). Combined, these results suggest higher OS and elevated glutathione metabolism in the hippocampus of epileptic patients.

### The transcriptome of TLE-HS hippocampi confirms higher glutathione metabolism and altered iron trafficking

**Fig. 3:**
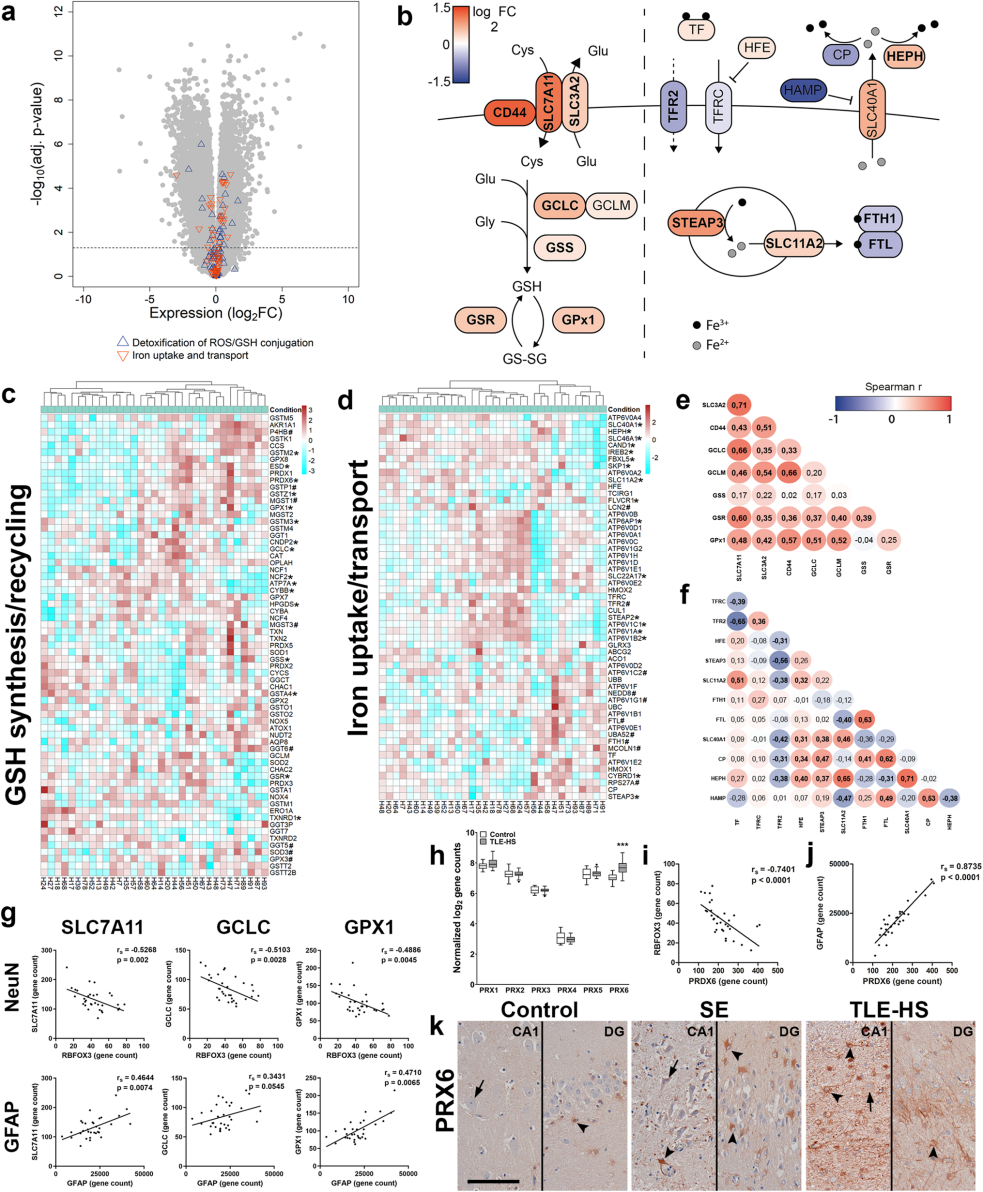
The transcriptome of TLE-HS hippocampus tissue confirms alterations in iron metabolism and glutathione metabolism, revealing strong correlations between genes of similar function, but also heterogeneity between patients. **a** Volcano plot of TLE-HS hippocampi compared to autopsy control tissue yielded 5937 upregulated and 6125 downregulated genes, many of them being involved in either detoxification of ROS and glutathione conjugation or iron uptake and transport. **b** A more focused visualization of principal components of glutathione synthesis/recycling (left) and iron uptake and transport (right). **c**, **d** Clustering of samples (Euclidean distance) using genes involved in glutathione recycling/synthesis and iron uptake/transport revealed heterogeneity between TLE-HS patients. **e** Correlation analysis revealed a strong correlation between glutathione synthesis/recycling genes, except GSS. **f** Correlation analysis of genes involved in iron uptake and transport revealed positive correlations between i.e. iron binding (FTH-1/FTL), iron export (HEPH/FPN1 [SLC40A1]), while iron uptake (TFR2/TF) was negatively correlated. **g** While NeuN [RBFOX3] was negatively correlated, GFAP correlated positively with xCT [SLC7A11], GCLC and GPX1. **h**–**j** Of the family of PRX proteins, PRX6 displayed strong upregulation in TLE-HS (**h**) and was negatively correlated with NeuN [RBFOX3] (**i**), but positively correlated with GFAP (**j**). **k** Immunohistochemical analysis of PRX6 revealed a predominantly astrocytic staining. Sections in k were counterstained with hematoxylin. **a** Dashed line represents a cut-off of Benjamini–Hochberg corrected *p* < 0.05. **b** bold = p < 0.05. **c**, **d** Euclidean distance clustering. Genes differentially expressed compared to control are indicated with *(upregulated, adjusted *p*-value < 0.05) and # (downregulated, adjusted *p*-value < 0.05), respectively. **e**, **f** Numbers represent Spearman *r* (*r*_s_) with bold numbers *p* < 0.05. **e**–**g**, **i**, **j** Spearman’s rank test. RNA sequencing: *n* = 11 control autopsy hippocampi vs. *n* = 33 TLE-HS hippocampi. SLC3A2 = 4F2 cell-surface antigen heavy chain, SLC7A11 = xCT, SLC11A2 = DMT-1, SLC40A1 = FPN-1

To validate the current findings and utilize the advantages of high-throughput analysis, transcriptomic data from TLE-HS hippocampi compared to autopsy control tissue was employed. In total, 6125 downregulated and 5937 upregulated genes were identified, containing a multitude of genes involved in functions related to antioxidant capacity and iron metabolism (Fig. 3a). Two sets of genes relating to different functional modules were sub-selected for analysis: 1. a combined list of genes involved in detoxification of ROS and glutathione conjugation and 2. genes involved in iron uptake and transport. Investigation of a subset of genes involved in glutathione metabolism revealed upregulation of cysteine import (xCT [SLC7A11], SLC3A2, CD44), glutathione synthesis (GCLC, GSS) as well as recycling (GSR) and glutathione-dependent peroxidation (GPx1). A subset of genes involved in iron metabolism revealed lower expression of iron uptake (TFR2), iron binding (FTH1, FTL) and iron export suppression (HAMP), while genes involved in endosomal iron reduction and export (STEAP3, DMT-1 [SLC11A2]) as well as cellular iron export (FPN-1 [SLC40A1], HEPH) were overexpressed compared to autopsy control tissue. Besides its endosomal function, DMT-1 located at the membrane (not depicted) could potentially also facilitate cellular iron uptake depending on the cell type (Fig. 3b). Visualization of genes involved in glutathione synthesis/recycling and iron uptake/transport revealed distinct subgrouping of TLE-HS cases (Fig. 3c, d). A correlation analysis of the subset of glutathione genes revealed positive correlations between genes involved in glutathione synthesis and recycling, except for GSS (Fig. 3e). Correlation analysis of the gene subset involved in iron uptake and transport revealed negative correlations between TFR2 and genes involved in intracellular iron uptake (HFE, STEAP3, SLC11A2/DMT-1) and cellular export (HEPH, CP, SLC40A1/FPN-1). Moreover, strongest positive correlations were detected i.e. in genes involved in iron binding (FTH-1, FTL) and iron cycling (HEPH, SLC11A2/DMT-1, SLC40A1/FPN-1) (Fig. 3f). To investigate the relationships between cytopathology and the transcriptional changes of glutathione metabolism, a correlation analysis was conducted of xCT [SLC7A11], GCLC and GPX1 with either the astrocyte marker GFAP or neuronal marker RBFOX3 (NeuN). All three genes correlated negatively with RBFOX3 (xCT [SLC7A11]: *r*_s_ = -0.5268, *p* = 0.002; GCLC: *r*_s_ = − 0.5103, p = 0.0028; GPX1: *r*_s_ = − 0.4886, *p* = 0.0045), while they correlated positively with GFAP (xCT [SLC7A11]: *r*_s_ = 0.4644, *p* = 0.0074; GCLC: *r*_s_ = 0.3431, *p* = 0.0545; GPX1: *r*_s_ = 0.4710, *p* = 0.0065) (Fig. 3g). Moreover, of the family of peroxiredoxins (PRX), we found specifically PRX6 to be upregulated in TLE-HS, which revealed negative correlation with RBFOX3 (*r*_s_ = − 0.7401, *p* < 0.0001) and positive correlation with GFAP (*r*_s_ = 0.8735, p < 0.0001) (Fig. 3h-j). This strong co-expression with GFAP was validated on tissue, revealing strong astrocytic PRX6 expression in SE and TLE-HS (Fig. 3k). These data validate substantial alterations in iron metabolism and suggest that predominantly astrocytes are responsible for antioxidant gene expression in hippocampi of epilepsy patients.

### Postictally increased magnetic susceptibility in epilepsy patients

**Fig. 4:**
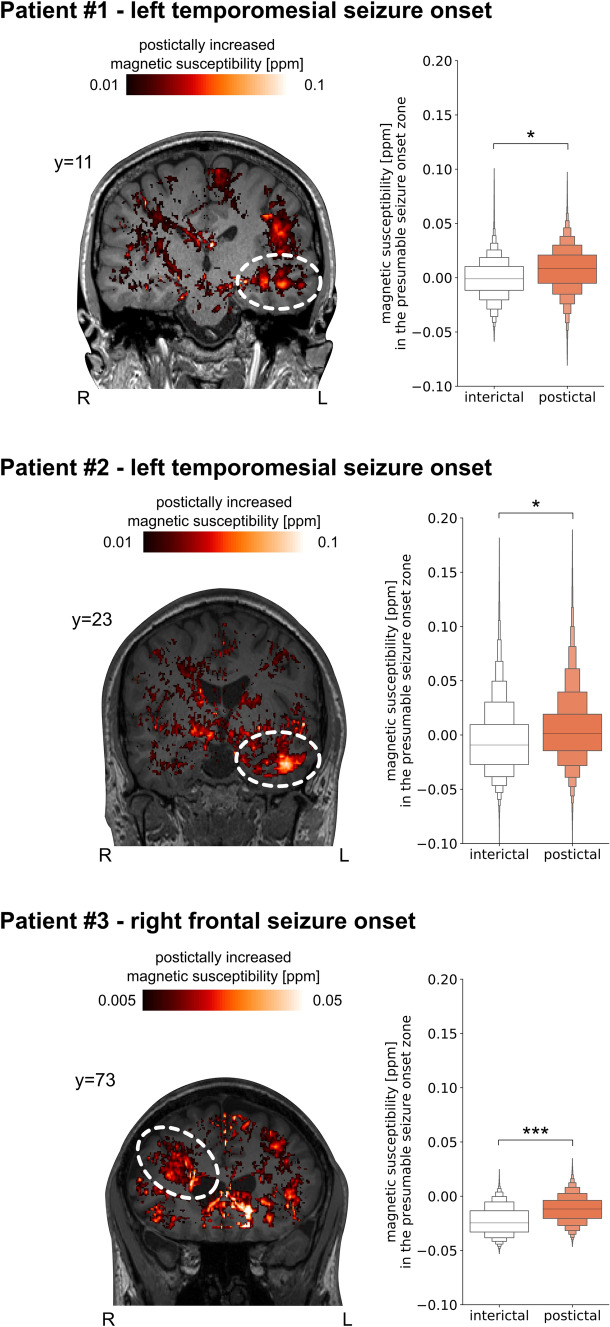
Postictal quantitative susceptibility in three epilepsy patients with focal seizures QSM scans of three epilepsy patients showed an increase in postictal magnetic susceptibility, indicative for an increased iron accumulation within the presumable seizure-onset zone. QSM difference images have been thresholded, visualized according to the indicated color scale, and superimposed on the structural T1-weighted image. The presumable seizure-onset zone is schematically indicated by white dashed ellipses. Voxelwise distributions were read out within manually demarcated masks of the seizure-onset zone, visualized as letter-value plots (midline indicating the median), and compared using Cohen’s d (***d > 0.8, *d > 0.2)

Evidence from experimental epilepsy models suggest seizure-dependent opening of the BBB, which is hypothesized to promote the extravasation and intracerebral accumulation of iron-rich blood components such as protein-bound iron. However, thus far, this hypothesis has not been tested in human subjects. To this end, the magnetic susceptibility of the electroencephalographically determined seizure-onset zone of three patients diagnosed with focal epilepsy were compared inter- and postictally. Patients #1 and #2 showed a temporomesial seizure onset as ascertained by ictal EEG, while patient #3 presented with a radiologically diagnosed focal cortical dysplasia type 2b located in the right frontal lobe (Fig. 4). In all three patients, a postictally (compared to interictally) increased magnetic susceptibility in the immediate vicinity of the presumable seizure-onset zone can be visually appreciated in the QSM difference image (Fig. 4). Comparisons of the voxel-wise magnetic susceptibility distributions showed higher postictal susceptibility values as compared to interictal susceptibility values within the seizure-onset zone with small effect sizes in the two temporomesial cases (Cohen’s d > 0.2) and a large effect size (Cohen’s d > 0.8) for patient #3. In conclusion, these results support higher iron accumulation in the seizure-onset zone after seizure activity.

### Prominent changes in iron accumulation and iron metabolism acutely after electrically induced SE and after development of spontaneous recurrent seizures in post-SE rats

**Fig. 5:**
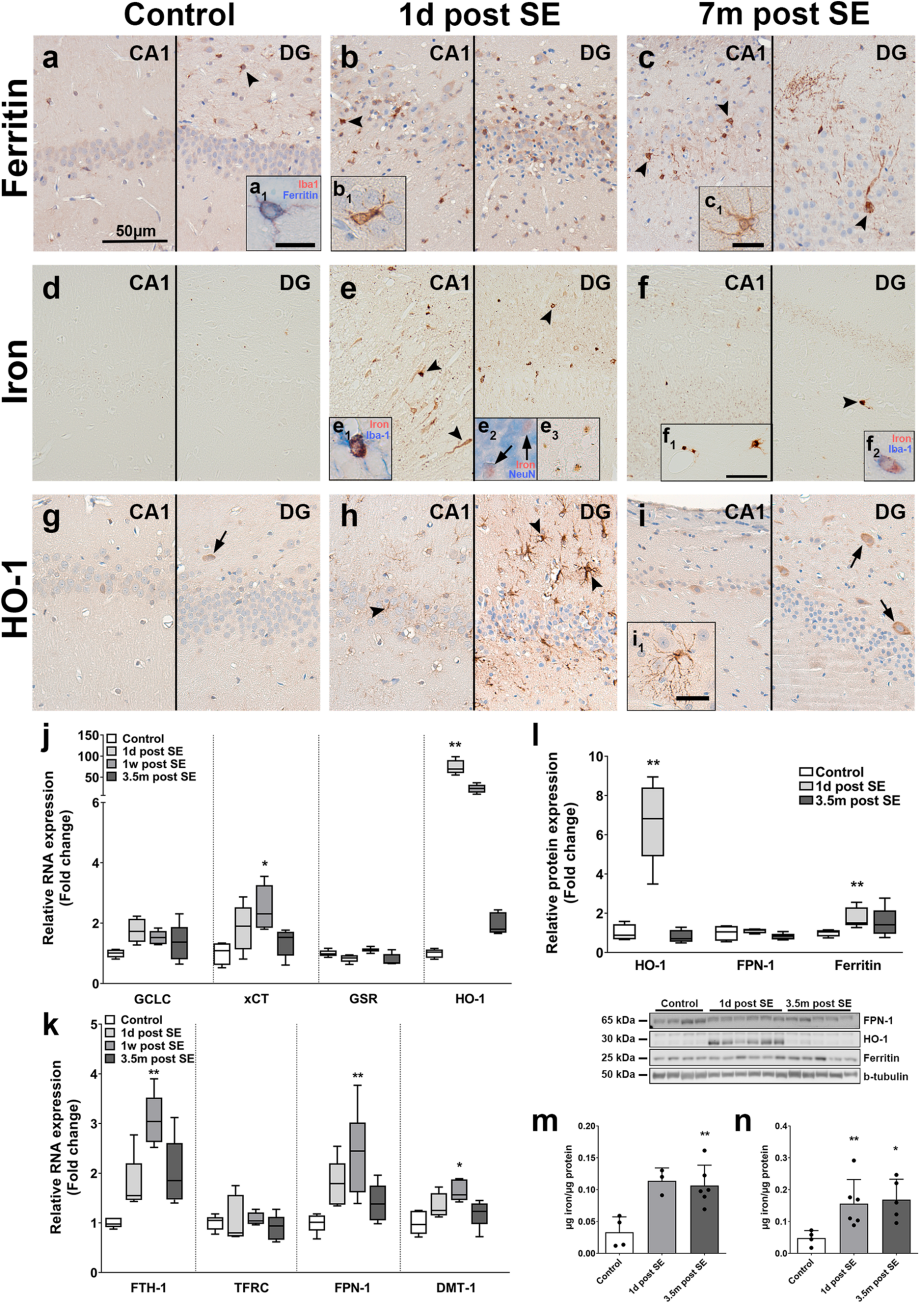
Hippocampal tissue of SE rats displays ferritin expression and iron accumulation in microglia, acute HO-1 expression in astrocytes and higher expression of OS response and iron metabolism genes after electrically induced status epilepticus. **a**–**c** Control rat hippocampus revealed ferritin expression in CA1 and DG in microglia (**a**, a_1_, arrowhead). Distribution of ferritin expression was not altered during the acute stage (1 day after electrically induced SE), being present in microglia with activated morphology and neurons. However, overall, more ferritin-positive microglia could be detected (**b**, b_1_, arrowhead). During the chronic phase (7 months post-SE), when rats have SRS, ferritin expression localized to microglia but also occasionally in astrocytes (c_1_) and the dendritic tree of neurons (c, arrowheads). **d**–**f** Iron in control hippocampi was detected in oligodendroglia and some neuronal nucleoli (**d**). Iron accumulation during the acute stage was markedly higher, accumulating in microglia (**e**, e_1_, arrowheads), neurons (e_1_, e_2_) and in the neuropil (e). In hippocampi of rats with SRS (chronic stage), iron could be detected in microglia (f_1_, f_2_) and in the neuropil (**f**). **g**–**i** HO-1 expression in control rats was restricted to few hilar interneurons (g, arrow). During the acute phase, strong expression of HO-1 was observed in astrocytes (h, arrowheads), which persisted in astrocytes with less reactive morphology during the chronic stage (**i**, i_1_). In addition, HO-1 expression in hilar neurons was elevated in animals suffering from SRS (i, arrows). **j**, **k** RNA expression in the PHC of TLE rats displayed most modulation during the latent phase with higher xCT, FTH-1, FPN-1 and DMT-1 expression. Moreover, HO-1 expression was higher during the acute stage as compared to control. **l** On the protein level, HO-1 and ferritin expression were higher during the acute stage compared to control, but not during the chronic stage. **m**, **n** Iron concentrations in the hippocampus (m) and the PHC (n) were higher in animals during the acute and chronic stages. Sections **a**–**c** and **g**–**i** were counterstained with hematoxylin. Scale bar = 50 µm in a, f_1_, 25 µm in i_1_, 10 µm in a_1_ (representative for b_1_, e_1_, e_2_, f_2_), c_1_; arrows = neurons, arrowheads = glia. **j**, **k** Kruskal–Wallis test followed by post hoc Dunn’s test. **l**–**n** Mann–Whitney *U* test. Data are expressed relative to expression observed in controls as Tukey box plots (**j**–**l**) or bar graphs with SD (**p**, **q**); *p < 0.05, **p < 0.01. **j**, **k** *n* = 5 (control, 1 day, 7 months) or *n* = 6 (1 week); **l**, **n** *n* = 4 (control), *n* = 6 (1 day) or *n* = 5 (3.5 months) animals per group. **m** *n* = 4 (control), *n* = 3 (1 day) or *n* = 6 (3.5 months) animals per group

To corroborate our findings from human tissue and better understand the time-course of the observed changes, RNA and protein expression of iron metabolism molecules as well as iron concentrations were measured in the hippocampus of post-SE rats at different stages after electrically induced SE (low magnification overviews can be found in Online resource 4a–i). Similar to humans, ferritin expression was predominantly found in microglia (arrowheads) of control animals (Fig. 5a, a_1_) and low HO-1 expression in few neurons (arrow), while very low iron reactivity could be detected in oligodendrocytes and occasionally neuronal nucleoli (Fig. 5d, g). During the acute phase (1 day post-SE), ferritin expression was evident in activated microglia (Fig. 5b, b_1_), while a lot of iron accumulated in microglia (Fig. 5e_1_), some neuronal nuclei (Fig. 5e_2_, e_3_) and in the neuropil (Fig. 5e). Moreover, HO-1 expression was markedly higher in glia (Fig. 5h). During the chronic phase (7 months post-SE), when animals had SRS, ferritin was detected in microglia, a few astrocytes as well as across the dendritic tree of some neurons (Fig. 5c). Iron accumulation was less pronounced than 1 day post-SE, but could still be detected in microglia (Fig. 5f_1_, f_2_) and the neuropil (Fig. 5f). HO-1 expression was still detected in not only astrocytes, but also CA1 neurons and hilar interneurons (Fig. 5i, i_1_).

RNA expression in the PHC of post-SE rats at different stages revealed higher xCT, FTH-1, FPN-1 and DMT-1 expression during the latent phase (1 week post-SE), while HO-1 was higher during the acute phase (1 day post-SE) (Fig. 5j, k) as compared to controls. In hippocampal subfields CA1 and DG higher xCT expression during the latent phase could also be found (Online resource 4 k). Higher HO-1 expression in the acute phase was found in both CA1 and DG, while HO-1 expression in the latent phase was higher only in the CA1 during the latent phase (Online resource 4 k, m). Lower expression of TFRC in the DG in the chronic phase was the only detectable difference in iron metabolism in the hippocampal subfields (Online resource 4 l, n). On protein level, HO-1 and FTH-1 expression in the PHC were higher during the acute phase (Fig. 5l) as compared to controls. Iron concentrations in hippocampal lysates were found to be higher in rats during the chronic phase (Fig. 5m), while lysates of the PHC revealed higher iron concentrations both during the acute and chronic phase (Fig. 5n). These results indicate that electrically induced SE triggers iron accumulation and promotes chronic changes in iron metabolism and OS in the hippocampus.

### Effects of ferric iron overload on epileptiform activity in acute hippocampal slices

**Fig. 6:**
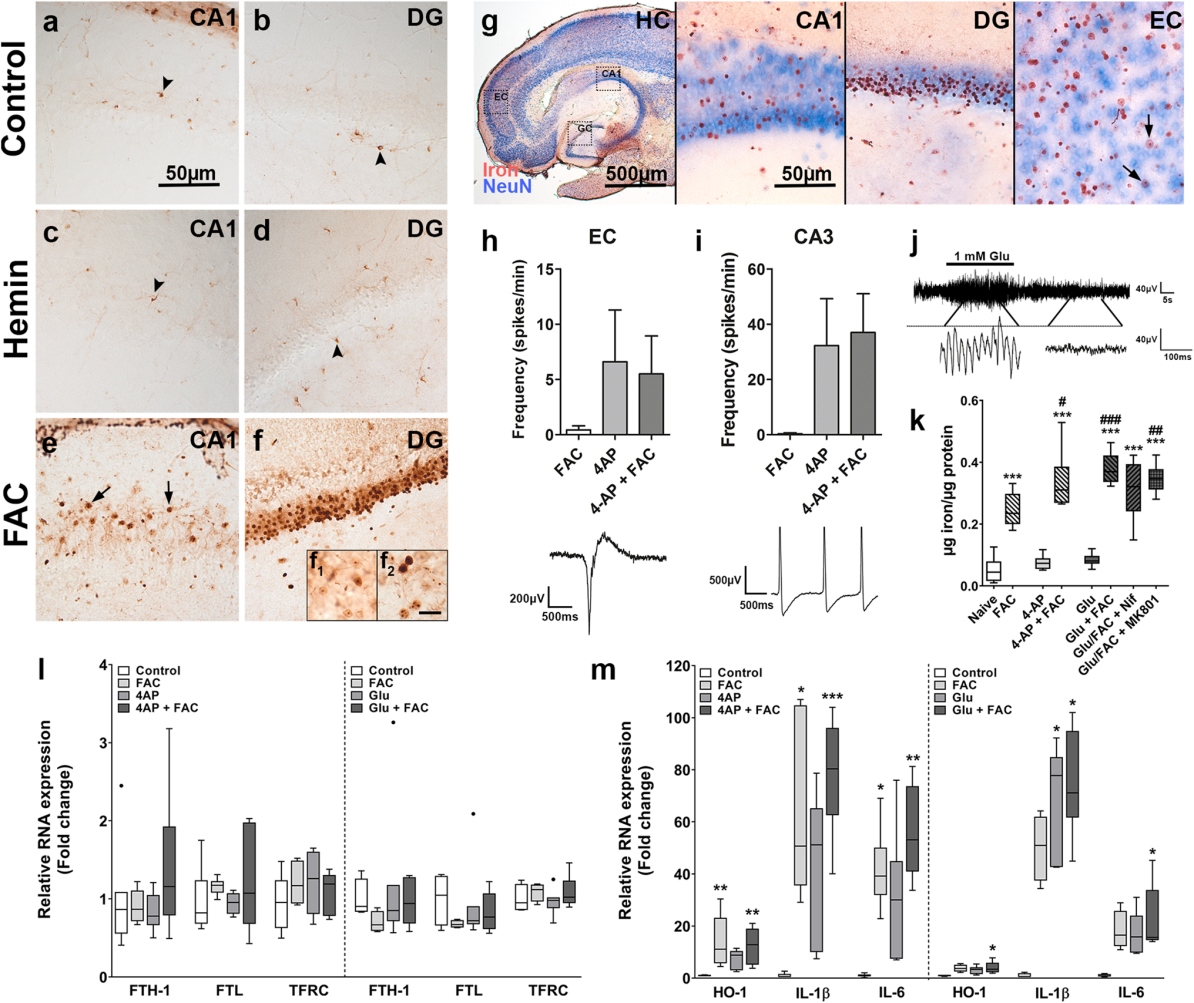
Iron accumulation upon exposure of acute hippocampal slices to ferric iron is exacerbated by epileptiform activity and induces pro-inflammatory gene expression. **a**, **b** Iron in the hippocampus of naive mouse slices could be detected in cells with oligodendroglial morphology (arrowheads) but was absent in neurons. **c**, **d** Exposure of mouse slices to hemin (10 μM) did not induce iron accumulation, revealing iron in cells with oligodendrocyte morphology similar to control (arrowheads). **e**, **f** In contrast, hippocampal slices exposed to FAC (200 μM) displayed marked iron accumulation in CA1 and DG neurons and their processes (arrows). **g** Co-labelling of iron with NeuN in FAC-treated slices revealed iron accumulation in a subset of neurons (EC, arrows). **h**, **i** The frequency of epileptiform discharges (example traces underneath) in the EC (**h**) and CA3 (**i**) did not differ between brain slices treated with 4-AP (100 μM) alone versus 4-AP/FAC. FAC alone did not induce any spontaneous activity. **j** Exposure of hippocampal brain slices to l-glutamate (Glu,1 mM)-induced brief paroxysmal activity for approximately 1 min after which activity returned back to baseline. **k** Quantification of total iron showed iron accumulation in brain slices exposed to FAC compared to untreated, naïve brain slices. Co-treatment with FAC plus either 4-AP or Glu led to an even higher iron accumulation in brain slices compared to FAC alone, that was not reduced by co-treatment with either 10 µM nifedipine or 20 µM MK801. **l** Assessment of RNA expression of hippocampal slices did not reveal modulation of FTH-1, FTL or TFRC after treatment with FAC, 4-AP, Glu or a combination. **(m)** In contrast, expression of HO-1, IL-1β and IL-6 was upregulated after treatment with FAC or FAC/4-AP, but not 4-AP alone (m, left). Treatment with Glu and Glu/FAC induced higher expression of HO-1, IL-1β and IL-6 (m, right). Scale bars: 500 µm in g, 50 µm in a (representative for **a**–**f**) and g (CA1 representative for DG, EC), 10 µm in f_2_ (representative for f_1_); arrows = neurons, arrowheads = glia. **l**, **m** Kruskal–Wallis test followed by post hoc Dunn’s test. Data are expressed relative to expression observed in controls as bar graphs with SD (**h**, **i**) or Tukey box plots (**k**–**m**); *p < 0.05, **p < 0.01, ***p < 0.001 versus control; ^#^*p* < 0.05, ^###^*p* < 0.001 vs. FAC. **h**, **i** *n* = 2 (FAC) or 4–5 (4-AP, 4-AP/FAC), mean from 2 to 3 electrodes per brain area (EC or CA3); **k** *n* = 8 slices from 4 animals per condition; **l,** **m** *n* = 6–7 slices from 3 to 4 animals per condition

To mimic the acute effect of seizure-mediated iron leakage and iron exposure on brain tissue, acute hippocampal brain slices were exposed to hemin or FAC for 1 h. Of note, 200 µM FAC correspond to 8.6–9.7 µg/mL iron assuming a reference range of 5–17.5 µg/mL serum iron, implying a physiologically relevant iron load upon BBB leakage. While iron reactivity in control and hemin-treated slices could be detected in cells with microglial and oligodendroglial morphology (arrowheads), FAC treatment led to prominent iron staining in neurons, particularly in neuronal processes as well as neuronal nuclei and nucleoli (Fig. 6a–g, arrows). Treatment with FAC did not induce spontaneous epileptiform activity in hippocampal slices nor did it exacerbate 4-AP-induced epileptiform activity (Fig. 6h–i). However, total iron concentration in hippocampal slices was elevated upon co-treatment with either 4-AP or l-glutamate (Glu, which induced brief paroxysmal activity in the slice, Fig. 6j) which was not reduced by the voltage-gated calcium channel (VGCC) inhibitor nifedipine or the *N*-methyl-d-aspartate (NMDA) receptor inhibitor MK801 (Fig. 6k). Immunohistochemically, no prominent visual differences between FAC and FAC/4-AP or FAC/Glu-treated slices could be detected (not shown). Measurement of RNA expression in hippocampal slices after exposure to FAC, 4-AP, Glu or a combination did not alter expression of iron metabolism genes (Fig. 6l). On the contrary, HO-1, IL-1β and IL-6 expression were induced by FAC, FAC/4-AP, Glu and Glu/FAC treatment, while 4-AP stimulation alone did not induce changes. Overall, co-stimulation of FAC combined with either 4-AP or Glu induced more robust changes (Fig. 6m). Finally, as marker for neuronal activation, we found higher cFOS expression in 4-AP and FAC/4-AP, and Glu/FAC-treated slices (Online resource 4j). These data indicate that ferric iron overload does not induce epileptiform activity but leads to activity-dependent iron uptake in neurons and amplifies upregulation of pro-inflammatory genes.

### Human fetal astrocytes in vitro resist chronic OS and iron overload, but respond with a pro-inflammatory phenotype

**Fig. 7:**
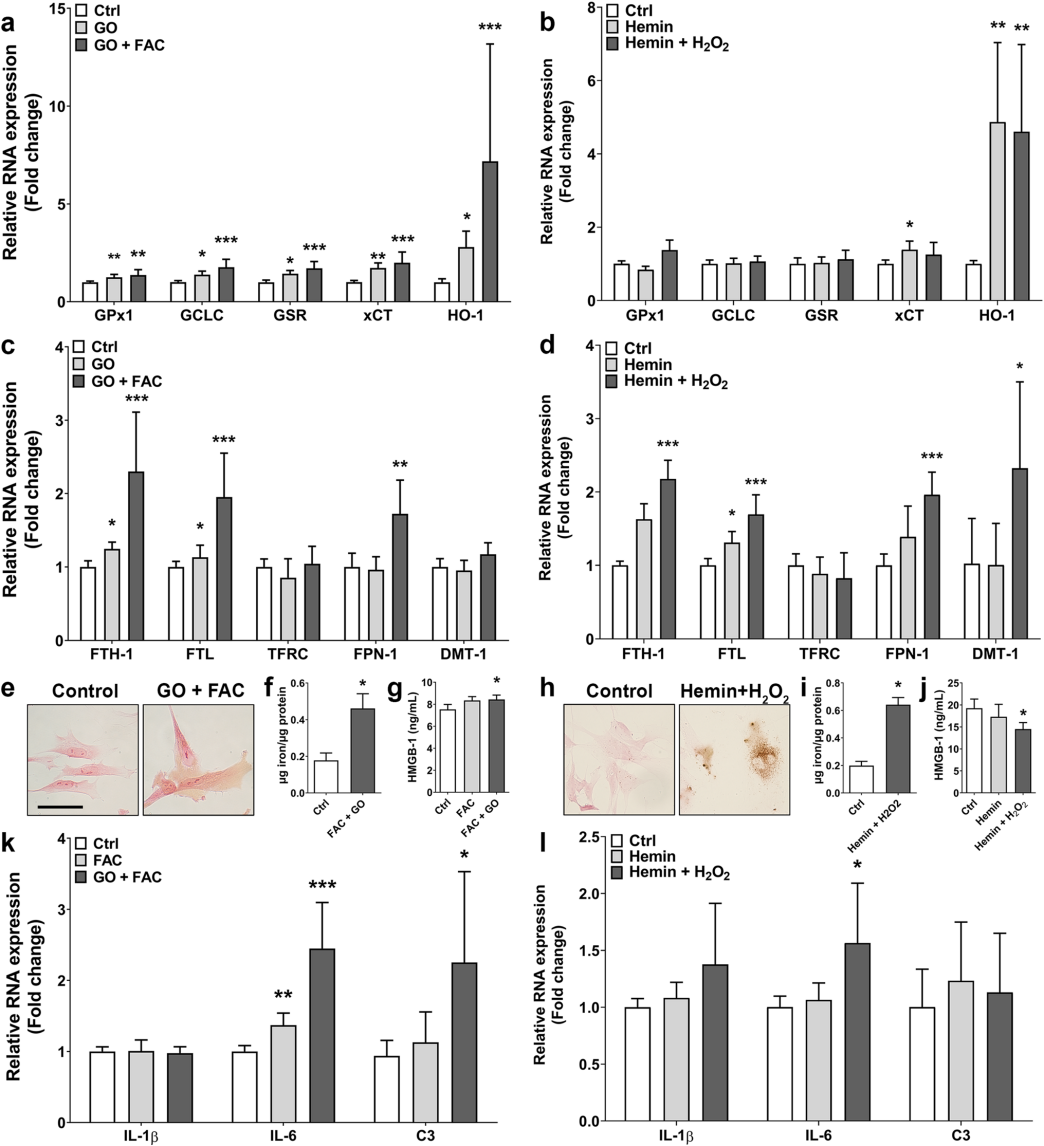
In vitro, human fetal astrocytes exposed to OS and iron induce glutathione metabolic genes and iron metabolism and promote a pro-inflammatory phenotype. **a**–**d** Human fetal astrocytes exposed to GO (2.5 mU) and FAC (200 μM) for 72 h displayed higher expression of glutathione genes GPx1, GCLC, GSR, xCT, HO-1 and FTH-1 and FTL than GO alone compared to control (**a**, **c**). Additionally, FPN-1 was exclusively higher in astrocytes exposed to GO and FAC (**c**). Fetal astrocytes exposed to hemin (10 μM) for 24 h only displayed upregulation of FTL while the combination of H_2_O_2_ (500 μM) with hemin induced the expression of GSR, HO-1, FTH-1, FPN-1, DMT-1 (**b**, **d**). **e**, **f** Fetal astrocytes exposed to GO/FAC for 24 h accumulate intracellular iron. **g** Stimulation of fetal astrocytes with GO/FAC, but not FAC alone, led to release of HMGB-1 into the culture supernatant. **h**, **i** Similar to FAC, exposure of fetal astrocytes to hemin-induced iron accumulation. **j** However, in contrast to FAC, HMGB-1 release into the supernatant was reduced upon co-treatment of hemin/H_2_O_2_. **k**, **l** RNA quantification revealed higher IL-6 expression upon treatment with FAC or GO/FAC. However, only FAC/GO induced the expression of C3 (k). Fetal astrocytes stimulated with hemin or hemin/H_2_O_2_ displayed solely upregulation of IL-6 upon hemin/H_2_O_2_ co-stimulation. Scale bars: 50 µm in e (representative for h). **a**, **b**, **g**, **j**, **k**, **l** Kruskal–Wallis test followed by post hoc Dunn’s test. **f**, **i** Mann–Whitney *U* test. Data are expressed relative to expression observed in controls as bar graphs with SD; *p < 0.05, **p < 0.01, ***p < 0.001. **a**, **b**, **k**, **l** *n* = 4 independent cultures in duplicates, **g** *n* = 5 independent cultures; **f**, *n* = 4 independent cultures; **j** *n* = 6 independent cultures. **e**, **h** Cells on coverslips were counterstained with Safranin O. Scale bar = 50 µm in (**e**)

Since prominent ferritin expression was found specifically in astrocytes in tissues from epilepsy patients, human fetal astrocytes were stimulated with OS and iron to further explore iron’s effect in vitro. Here, two paradigms were chosen: 1. modeling chronic OS and iron overload by stimulating with GO and FAC for 72 h and 2. modeling acute seizure-induced extravasation of iron-rich blood components combined with acute OS via hemin/H_2_O_2_ for 24 h. RNA expression of glutathione metabolic genes GPx1, GCLC, GSR and xCT as well as HO-1 was higher in fetal astrocytes stimulated with GO compared to control and this difference was higher when cells were co-stimulated with FAC (Fig. 7a). Stimulation with hemin-induced HO-1 and xCT expression while co-stimulation with H_2_O_2_ had no additional effect (Fig. 7b). Expression of FTH-1 and FTL was higher upon FAC and GO/FAC stimulation, while FPN-1 was only elevated compared to control in cells exposed to GO/FAC (Fig. 7c). Similar changes were observed for hemin stimulation, which led to upregulation of FTL. Co-treatment of hemin/H_2_O_2_ induced FTH-1 FTL, FPN-1 and DMT-1 upregulation (Fig. 7d). Time-dependent changes in RNA expression of glutathione and iron metabolism genes in response to FAC, GO or H_2_O_2_ in astrocytes were investigated as well (Online resource 5a-f). These experiments revealed prominent changes in iron metabolism genes predominantly in response to OS, not iron overload (Online resource 5b, d, f). Positive Perl’s iron stain and quantification of iron in fetal astrocytes exposed to GO/FAC or hemin/H_2_O_2_, indicated an uptake of iron (Fig. 7e, f, h, i). Moreover, without compromising cell viability (Online resource 5i, j), HMGB-1 in the supernatant was higher in chronic GO/FAC stimulation, while it was lower in hemin/H_2_O_2_-treated astrocytes (Fig. 7g, j). Since astrocytes are important mediators of neuroinflammation in TLE-HS, expression of pro-inflammatory mediators upon FAC and GO/FAC or hemin and hemin/H_2_O_2_ were assessed as well. RNA expression of interleukin 1 β (IL-1β) was not different and IL-6 expression was increased in both conditions with FAC alone and GO/FAC. Contrary, C3 expression was specifically induced in cells exposed to GO/FAC (Fig. 7k). Hemin treatment for 24 h alone did not have an effect while hemin/H_2_O_2_ treatment induced IL-6 expression (Fig. 7l), although IL-6 in cell culture supernatant was not different (Online resource 5 g, h). To investigate if proinflammatory gene expression in response to iron accumulation in astrocytes is complemented by other factors resulting from BBB dysfunction that also co-localize in TLE-HS hippocampi (Online resource 2 m–p), human fetal astrocytes were stimulated with BSA and HSA. IL-1β was elevated after 24 h and C3 and COX-2 after 48 h stimulation with BSA while HO-1 did not change (Online resource 5 k, l). A similar trend was observed for 24 h stimulation with HSA. Matrix metalloproteinase 9 (MMP9) served as positive control, as expression of MMP9 was previously demonstrated to be induced by albumin.

Finally, SHSY5Y neuroblastoma cells were investigated to mimic the neuronal response to OS and iron. GO stimulation induced xCT and FTL expression, whereas GO/FAC induced GPx1, GSR, xCT, HO-1, FTH-1, FTL and lowered TFRC expression (Online resource 6a, b). Exposure to GO/FAC and hemin/H_2_O_2_ led to iron uptake and elevated HMGB-1 secretion into the supernatant, while compromising cell viability only when stimulated with hemin/H_2_O_2_ (Online resource 6c-h). Lastly, while not compromising viability, stress-related alterations like reduced electron density and occasional calcium pits, without dramatic morphological alterations in SHSY5Y mitochondria could be observed after FAC challenge (Online resource 6i). In conclusion, astrocytes in vitro are capable of taking up and detoxifying iron upon ferric iron challenge, but secrete pro-inflammatory factors, while neurons secrete damage-associated signaling molecules and display signs of mitochondrial stress.

## Discussion

The presented data demonstrate that brain tissue from epilepsy patients and rodent epilepsy models respond with iron accumulation as well as changes in iron and glutathione metabolism. Moreover, seizures appear to promote iron accumulation in brain tissue of epilepsy patients in the seizure-onset zone, while ictogenic substances increase iron uptake into acute hippocampal slices. Finally, astrocytes seem to acquire the capacity to sequester iron, while not only upregulating their antioxidant capacity but also producing pro-inflammatory signals.

### Nuclear iron accumulation and lipid peroxidation in neurons in SE and TLE-HS brain tissue implicate iron in cell stress

A prerequisite for iron-catalyzed neuronal dysfunction is the accumulation of intracellular iron. Indeed, iron accumulation in CA1 neurons of SE and TLE-HS tissue could be found specifically in neuronal nuclei and nucleoli. Iron in neuronal nuclei was previously demonstrated upon ultrastructural analysis in the rat brain, supposedly playing important physiological roles e.g. in the synthesis of ribosomal RNA [31, 48, 64, 92]. Notably, very strong iron accumulation in neuronal nuclei was detected in TLE-HS and SE in pyknotic neurons. Since pyknosis is a feature of degenerating neurons, this finding indicates that nuclear iron accumulation accompanies neuronal cell death in TLE-HS. Furthermore, ferritin was not expressed in neurons, indicating a low iron storage capacity. In agreement with this finding, elevated nuclear iron accumulation was previously found in degenerating neurons in the context of hereditary iron storage disorders and Alzheimer’s disease [86, 94]. Although total neuronal iron load in SE and TLE-HS tissue was low, the DAB-enhanced Perl’s method detects Fe^3+^ [47]. Hence, the visualized iron likely represents non-reactive, protein-bound Fe^3+^ iron. Unbound, soluble Fe^2+^, which represents the more Fenton-reactive pool, could also accumulate and more readily react with ROS in neurons.

Higher 4-HNE reactivity was detected in neurons in SE and TLE-HS tissue. Additionally, high 4-HNE adducts could be detected in the CSF and overexpression of glutathione metabolic enzymes in the hippocampus of TLE-HS patients, indicating a more global state of OS in the brain. 4-HNE represents a marker of lipid peroxidation, a process previously implicated in iron-catalyzed regulated necrosis, termed ferroptosis [16, 80]. Ferroptosis is prevented via system Xc^−^-mediated cysteine import, the key metabolite in glutathione synthesis required for lipid-ROS detoxification by lipid peroxidases. In turn, system Xc^−^ is a glutamate/cysteine antiporter, hence elevated extracellular glutamate concentrations in epilepsy could not only promote excitotoxicity but also facilitate ferroptosis [80]. While the exact cell-death mechanism of pyknotic neurons in TLE-HS is unclear, previous studies demonstrated that specifically hilar GABAergic neurons, a neuronal subtype whose dysfunction is supposed to play a pro-epileptogenic role in patients with TLE and animal models of acquired epilepsy, seem to be highly sensitive to ferroptosis [16, 32, 66, 90]. A protective response of the brain might require upregulation of the catalytic subunit of system Xc^−^, xCT, for sufficient glutathione synthesis. This is in line with the detected overexpression of glutathione metabolism on total hippocampal RNA and protein in TLE-HS tissue. Moreover, only a fraction of 4-HNE-positive neurons exhibited pyknotic morphology, suggesting elevated lipid peroxidation does not automatically predestine cells for necrosis. Besides 4-HNE, neurons also showed high expression of HO-1, an acute stress protein whose transcription is regulated by antioxidant defense systems like the Nrf-2 signaling pathway [53]. Importantly, HO-1 represents a key enzyme in the acute antioxidant response releasing iron in the process [72]. Consequently, acute HO-1 expression is involved in detoxification, while chronic Nrf-2-dependent HO-1 overexpression induces iron release [97] and paradoxically was shown to accelerate oxidative damage and ferroptosis [38, 72, 79]. Thus, in addition to extrinsic seizure-mediated iron ingress via BBB leakage, neurons might also be confronted with iron overload via intrinsic enzyme-mediated iron release.

### Iron accumulation and ferritin expression in glia implicate increased iron-binding capacity of astrocytes in SE and TLE-HS tissue

A principle finding in SE and TLE-HS brain tissue was a prominent shift in the localization of ferritin expression from microglia and oligodendrocytes in autopsy control tissue to markedly higher expression in astrocytes. Microglia were shown to upregulate ferritin expression in experimental epilepsy models [23, 29]. In addition, astrocytes appear to dynamically acquire the capacity to sequester iron in SE and TLE-HS. Interestingly, astrocytic ferritin overexpression was accompanied by a decrease in total ferritin gene and protein expression in TLE-HS hippocampi. One explanation for the reduction of total ferritin might be that iron sequestration via microglia is dysfunctional or overloaded in epilepsy, either because of higher brain iron load due to seizures or altered microglial iron homeostasis. In this context, recent developments in single cell transcriptomics revealed that microglial subtypes show more distinct changes in homeostatic functions during pathological situations, which could include iron metabolism [44]. This shift in iron handling from microglia to astrocytes is supported by a previous report studying multiple sclerosis brains, which revealed a similar function for reactive astrocytes as iron-storing cells [62]. Besides iron and ferritin, TLE-HS tissue also displayed lower RNA expression of TFRC and higher expression of DMT-1 in epileptogenic tissue. TFRC is involved in transferrin-dependent Fe^3+^ iron uptake, while DMT-1 facilitates endosomal iron export, but also direct transmembrane entry of Fe^2+^ into cells. Immunohistochemistry of TFRC and DMT-1 revealed low neuronal TFRC and higher TFRC and DMT-1 expression in astrocytes. DMT-1 shuttles Fe^2+^ while TFRC transports transferrin bound Fe^3+^ [30], thus, indicating either a shift in the brains Fe^2+^/Fe^3+^ ratio or changes in iron handling such as an increased need to rapidly take up Fe^2+^ or metabolize endosomal Fe^2+^. Strong DMT-1 and TFRC expression in astrocytes suggests higher glial uptake of iron, while overall lower TFRC might be explained by neuronal loss but also reduced expression including other cell types such as endothelial cells. Additionally, higher FPN-1 RNA expression might indicate enhanced cellular iron export [13]. The intimate interaction of astrocytes with the BBB and their consequent improved ability to control iron flux as compared to microglia could be another explanation for the shift of iron sequestration to astrocytes in epilepsy [46, 87, 91]. Interestingly, strong TFRC expression in punctate, astrocytic end-feet around the microvasculature could be detected, which might indicate a shift in control over iron shuttling from endothelium to astrocytes in epilepsy. In addition to SE/TLE-HS tissue, TBI and stroke lesions were studied, since brain tissue from both injuries typically exhibit brain iron overload due to hemorrhages. Notably, iron accumulation and ferritin expression in TBI and stroke lesions was detected in macrophages/microglia acutely, while astrocytes overexpressed ferritin in older lesions. This further confirms astrocytes to be crucial to iron sequestration during chronic iron exposure due to damaged/dysfunctional BBB or increased intraparenchymal iron release of brain cells.

### The transcriptome of TLE-HS hippocampi reveals higher glutathione metabolism and altered iron trafficking

The collected transcriptomic data suggest higher antioxidant capacity, while simultaneously reducing iron import and promoting iron secretion, overall confirming the initial findings. FPN-1-mediated iron secretion is hepcidin (HAMP) and redox dependent [14], mainly mediated by the ferroxidases hephaestin (HEPH) or ceruloplasmin (CP) [30]. CP was shown to be expressed predominantly in astrocytes and its lack thereof promoting astrocytic iron accumulation in aceruloplasminemia [34, 36, 55]. Thus, CP downregulation might promote iron retention specifically in astrocytes. On the other hand, HAMP downregulation, which promotes FPN-1 internalization [49], and HEPH overexpression support increased iron secretion in other cell types. Interestingly, the transcriptomic data also revealed that of the family of antioxidant peroxiredoxins, solely PRX6 was found to be upregulated in TLE-HS hippocampi. PRX6 constitutes the only glutathione-dependent peroxiredoxin that has high affinity for lipid hydroperoxides [17, 65]. Thus, the strong expression of PRX6 in astrocytes supports their high glutathione-dependent antioxidant capacity in TLE-HS. Notably, RNA sequencing showed a large heterogeneity between TLE-HS cases revealing distinct sub-clusters. Factors contributing to this heterogeneity between patients might include i.e. age, seizure frequency, epilepsy duration, cellular composition or last seizure before hippocampal resection.

### Ictal activity increases the magnetic susceptibility of brain tissue in the seizure-onset zone of epileptic patients

The analysis of magnetic susceptibility by means of QSM showed a postictal increase in susceptibility within (but not strictly limited to) the presumable seizure-onset zone in patients. In concordance with the current literature [70], this change in magnetic susceptibility might indicate an increased accumulation of brain iron temporally and spatially associated with the epileptic seizure [39]. Notably, magnetic susceptibility of Fe^2+^ and Fe^3+^ were shown to be similar, thus not allowing a precise discrimination between oxidation states [15]. Current evidence for acute BBB dysfunction and consequent iron influx upon brain insults such as TBI or SE is strong, while prolonged BBB opening was demonstrated to play a role in epileptogenesis and aggravation of the epileptic condition [84]. Moreover, brain intrinsic alterations in iron handling such as HO-1-mediated iron release or also iron release from iron-sulfur clusters in stressed mitochondria might impact the seizure-associated increase in QSM signal [56]. The presented QSM data clearly demonstrate an increased susceptibility as a consequence of seizure activity in vivo, while data from epilepsy brain tissue suggests alterations in iron homeostasis temporally independent of the seizure. This finding likely represents a symptom of ongoing BBB dysfunction in combination with dysfunctional clearance of postictal iron. Here, accumulated iron could contribute to increased excitability by impairing astrocytic function (e.g. reduced potassium buffering) or by stimulating neuroinflammation, as supported by the in vitro data. Accordingly, iron itself might not be ictogenic per se, but affect the progression of epilepsy by lowering the seizure threshold over time.

### Strong iron accumulation upon electrically induced SE and spontaneous recurrent seizure activity

Post-SE rats revealed that the relative iron load was not different between the acute and chronic phase, whereas immunohistochemical staining revealed much stronger iron retention during the acute phase. This finding could be explained by the difference in Fe^2+^/Fe^3+^ ratio since the method for measuring total iron detects both ionization states, whereas the Perl’s staining only detects Fe^3+^. Based on these differences, it could be assumed that more Fe^2+^ is present during the chronic stage, potentially mediated by an altered brain redox state. Ferritin expression was almost exclusively found in microglia and only occasionally in astrocytes when SRS occurred, though at much lower numbers than in human brain tissue. Thus, higher FTH-1 expression during the latent stage might be attributable to activated microglia, as also shown by previous studies, even before the onset of SRS [23, 29]. Here, FTH-1 upregulation was detected also after the development of SRS, when neuronal loss is minimal, implicating altered iron metabolism in the pathogenesis and progression of epilepsy [23]. Another contributing factor to the increase in iron during the acute stage might also be HO-1, which was strongly overexpressed by reactive astrocytes. In contrast to tissue iron, RNA expression of iron metabolic genes and xCT was highest in the latent phase. Altogether, these data suggest a strong iron influx acutely post-SE, followed by iron metabolism/removal in the latent stage and, finally, chronic, low iron influx after the development of SRS. Contrary to human tissue, astrocytes appear to play a less important role in iron sequestration in this model which may be explained by the difference in i.e. seizure focus, epilepsy duration and differences in astrocyte competence in handling iron between rodents and humans [96]. Nevertheless, post-SE brain tissue confirmed profound iron accumulation and changes in iron metabolism.

### In vitro stimulation of hippocampal brain slices and primary astrocytes with ferric iron implicates seizure-mediated neuronal iron uptake and production of pro-inflammatory mediators in epileptogenesis

In human and post-SE tissue iron accumulation was associated with seizure activity, but whether seizures also specifically promote neuronal iron uptake and/or iron evokes epileptiform activity remained an open question. Although previous reports demonstrated epileptogenic potential of intracortical iron injections in experimental animals, we did not find evidence for acute ictogenic properties of ferric iron in hippocampal slices [35, 89]. However, neurons in acute hippocampal slice preparations stimulated with FAC revealed neuronal iron uptake primarily into nuclei and nucleoli, further pinpointing the nucleus as potential site of neuronal iron storage. These findings support that neurons could rapidly take up ferric iron upon excessive, local exposure as also seen in SHSY5Y cells. Moreover, SHSY5Y cells also displayed altered mitochondrial ultrastructure without impaired viability upon FAC exposure. Assuming BBB leakage, iron accumulation in neurons of TLE-HS patients is probably a slow, chronic process that is likely accompanied and influenced by other epileptogenic events such as altered redox status, mitochondrial dysfunction, neuroinflammation, etc. Since Fe^3+^-citrate is known to be a substantial component of blood [26, 54], this way of iron exposure could be feasible. Additionally, seizure-mediated dysfunction of iron clearance and metabolism in brain cells might induce iron accumulation. Hence, we conclude that iron accumulation represents a consequence of seizures. The fact that hemin treatment did not lead to neuronal iron uptake indicates the importance of the iron-donating molecule as iron bound to the protoporphyrin ring of hemin requires enzymatic degradation via HO-1 for release [72]. While HO-1 RNA overexpression could be observed across all models, iron release from heme in hippocampal slices might have required longer incubation times.

Interestingly, stimulation of acute brain slices with epileptiform stimulants Glu or 4-AP plus FAC led to higher uptake of iron. Mechanistically, repetitive stimulation of the NMDA receptor during epileptiform activity could lead to increased DMT-1-dependent or direct NMDA-facilitated uptake of iron into neurons [9, 59]. Additionally, VGCCs, which are also implicated in epilepsy [95], were shown to facilitate neuronal iron uptake [21]. Using inhibitors of VGCCs and NMDA receptors we could not detect a reduction of iron uptake upon treatment with Glu, suggesting other, possibly unspecific routes of entry. Of note, while no ictogenic propensity of FAC was detected on the hippocampal network level, iron was shown to be involved in modulating synaptic excitability in physiological conditions at the cellular level [88]. An additional finding in hippocampal slices was the induction of pro-inflammatory cytokine expression upon FAC stimulation and an exacerbation of this effect when paired with 4-AP or Glu. In summary, although iron is not acutely ictogenic in hippocampal slices in the present study, it can drive neuronal iron uptake and promote expression of pro-inflammatory factors, which could reinforce ictogenesis and promote neuronal dysfunction.

Astrocyte cultures stimulated with hemin/H_2_O_2_ (mimicking acute extravasation of blood components as in epileptogenic injuries) displayed iron uptake and higher RNA expression of iron metabolic genes required for handling excess iron. In contrast, stimulation with FAC/GO (mimicking altered redox balance and iron metabolism in the chronic epileptogenic brain) induced expression of ferritin and FPN-1, but also glutathione synthesis enzymes. Moreover, HMGB-1 release coupled to higher expression of IL-6 and C3 indicate that chronic iron coupled to OS might promote neuroinflammation. Elevated secretion of HMGB-1 could also be found in SHSY5Y cells treated with FAC/GO, but in contrast to astrocytes also hemin/H_2_O_2_. Importantly, HMGB-1 disulfide formation is facilitated by OS, which can activate the toll-like receptor 4 (TLR4)-HMGB-1 axis in neurons promoting hyperexcitability, excitotoxicity and seizures [10, 33, 43]. Thus, inflammatory changes in astrocytes coupled to secretion of damage-associated molecular patterns such as HMGB-1 might be associated with astrocytic iron accumulation in epileptogenic brain tissue. Importantly, in conjunction with the proposed iron-catalyzed exacerbation of ROS, astrocytic expression of antioxidant, iron metabolism and pro-inflammatory genes was higher when co-treated rather than with ROS or iron alone. Another link between antioxidant and pro-inflammatory signaling might be the overexpression of PRX6 in astrocytes identified from transcriptomics since PRX6 possesses phospholipase A_2_ activity [17] which could be involved in the synthesis of inflammatory lipid mediators. Interestingly, previous studies demonstrated that pro-inflammatory activation can promote iron-buffering and resistance to OS in astrocytes. Thus, pro-inflammatory gene expression might reflect a homeostatic mechanism which, when activated chronically, could ultimately contribute to the progression of the disease [41, 60, 63]. In addition, other blood-borne factors such as albumin could reinforce the iron-mediated pro-inflammatory gene expression we detected and act synergistically as a result of BBB compromise in epilepsy to activate glia.

## Conclusion

In conclusion, iron influx is likely due to a compromised BBB and changes in intracellular iron handling in chronic TLE-HS and epilepsy models. While the frequency and duration of seizures probably are important, astrogliosis and subsequent BBB dysfunction specific to TLE-HS might be essential for the altered iron accumulation observed. Moreover, the QSM data suggest higher iron load associated with seizures of various etiology. While iron itself does not trigger seizures, its intracellular accumulation is facilitated by paroxysmal activity. Finally, besides higher antioxidant and iron-binding capacity, astrocytes exposed to OS and iron acquire a potentially detrimental pro-inflammatory phenotype. Hence, strategies to reduce iron uptake into susceptible neuronal subtypes as well as into astrocytes, thereby reducing pro-inflammatory responses, might pose novel therapeutic options in epilepsy. While antioxidant treatment targeting Nrf-2 was shown to be beneficial in epilepsy and epileptogenesis, approaches more specifically inhibiting iron-catalyzed lipid peroxidation and ferroptosis have proven beneficial in experimental epilepsy models and might aid to identify novel therapeutic avenues [40, 42, 93]. However, more insights into the treatment window and the exact mechanisms and markers of iron-mediated neuronal damage are urgently needed.

## Supplementary Information

Below is the link to the electronic supplementary material.

Supplementary file1 (PDF 2603 kb)

## Data Availability

The datasets generated and analysed during the current study are available on the European Genome-phenome Archive (EGA) data repository. The EGA can be found at ega-archive.org.

## Abbreviations

4-AP: 4-Aminopyridine
4-HNE: 4-Hydroxynonenal
ACSF: Artificial cerebrospinal fluid
AEC: 3'-Amino 9'-ethylcarbazole
AP: Alkaline phosphatase
BBB: Blood–brain barrier
BCA: Bicinchoninic acid assay
C1ORF43: Chromosome 1 open reading frame 43
CA1: Cornu ammonis 1
CP: Ceruloplasmin
CSF: Cerebrospinal fluid
CycA: Cyclin A
DAB: Diaminobenzidine
DG: Dentate gyrus
DMT-1: Divalent metal transporter 1 (SLC11A2)
EC: Entorhinal cortex
EF1-α: Elongation factor 1 α
FAC: Ferric ammonium citrate
FCS: Fetal calf serum
FPN-1: Ferroportin 1 (SLC40A1)
FTH-1: Ferritin heavy chain 1
FTL: Ferritin light chain
GAPDH: Glyceraldehyde 3-phosphate dehydrogenase
GCLC: Glutamate cysteine ligase catalytic subunit
GFAP: Glial fibrillary acidic protein
Glu: l-glutamate
GO: Glucose oxidase
GPx1: Glutathione peroxidase 1
GSR: Glutathione disulfide reductase
HMGB-1: High-mobility group box 1
HO-1: Heme oxygenase 1
HRP: Horse radish peroxidase
Hrpt: Hypoxanthine phosphoribosyltransferase 1
Iba-1: Ionized calcium-binding adapter molecule 1
IHC: Immunohistochemistry
IRS: Immunoreactivity score
MEA: Multi-electrode arrays
MTT: 3-(4,5-Dimethylthiazol-2-yl)-2,5-diphenyl tetrazolium bromide
NADPH: Nicotinamide adenine dinucleotide phosphate
Nrf-2: Nuclear factor erythroid 2 like 2
OS: Oxidative stress
PBS: Phosphate buffered saline
PCR: Polymerase chain reaction
PHC: Parahippocampal cortex
PLL: Poly-l-lysine
PRX6: Peroxiredoxin 6
PV: Parvalbumin
PVDF: Polyvinylidene difluoride
QSM: Quantitative susceptibility mapping
ROS: Reactive oxygen species
SE: Status epilepticus
SRS: Spontaneous recurrent seizures
TBI: Traumatic brain injury
Tbp: TATA box-binding protein
TBS-T: Tris-buffered saline with 0.1% Tween20
TFRC: Transferrin receptor 1
TLE-HS: Temporal lobe epilepsy with hippocampal sclerosis
TMM: Trimmed mean of M-values
xCT: Glutamate/cysteine antiporter system Xc^−^ light subunit (SLC7A11)
