# Supplementary material for: Seizure-mediated iron accumulation and dysregulated iron metabolism after status epilepticus and in temporal lobe epilepsy
Source: Acta Neuropathol. 2021 Jul 22;142(4):729–59. doi: 10.1007/s00401-021-02348-6 (PMC8423709; doi:10.1007/s00401-021-02348-6)
Supplement: Supplementary file 1 — Supplementary file1 (PDF 2603 kb) [file 401_2021_2348_MOESM1_ESM.pdf]

Online resources (Acta Neuropathologica)

SEIZURE-MEDIATED IRON ACCUMULATION AND DYSREGULATED IRON METABOLISM AFTER STATUS EPILEPTICUS AND IN TEMPORAL LOBE EPILEPSY

Till S. Zimmer[1], Bastian David[2], Diede W.M. Broekaart[1], Martin Schidlowski[2,3], Gabriele Ruffolo[4], Anatoly Korotkov[1], Nicole N. van der Wel[5,6], Peter C. van Rijen[7], Angelika Mühlebner[1,8], Wim van Hecke[8], Johannes C. Baayen[9], Sander Idema[9], Liesbeth François[10], Jonathan van Eyll[10], Stefanie Dedeurwaerdere[10], Helmut W. Kessels[11], Rainer Surges[2], Theodor Rüber[2], Jan A. Gorter[11], James D. Mills[1,12,13], Erwin A. van Vliet[1,11,*], Eleonora Aronica[1,14,*]

[1]Amsterdam UMC, University of Amsterdam, Department of (Neuro)Pathology, Amsterdam Neuroscience, Amsterdam, the Netherlands
[2]Department of Epileptology, University Hospital Bonn, Bonn, Germany
[3]German Center for Neurodegenerative Diseases (DZNE), Bonn, Germany
[4]Department of Physiology and Pharmacology, laboratory affiliated to Istituto Pasteur Italia, University of Rome Sapienza, Rome, Italy
[5]Amsterdam UMC, University of Amsterdam, Department Cell Biology and Histology, Amsterdam, the Netherlands
[6]Amsterdam UMC, University of Amsterdam, Department Electron Microscopy Center Amsterdam, Amsterdam, the Netherlands
[7]University Medical Center Utrecht, Brain Centre, Rudolf Magnus Institute for Neuroscience, Department of Neurosurgery, Utrecht, the Netherlands
[8]University Medical Center Utrecht, Department of Pathology, Utrecht, the Netherlands
[9]Amsterdam UMC, Vrije Universiteit Amsterdam, Department of Neurosurgery, Amsterdam Neuroscience, Amsterdam, the Netherlands
[10]Neurosciences Therapeutic Area, UCB Pharma, Braine-l'Alleud, Belgium
[11]University of Amsterdam, Swammerdam Institute for Life Sciences, Center for Neuroscience, Amsterdam, the Netherlands
[12]Department of Clinical and Experimental Epilepsy, UCL, London, United Kingdom
[13]Chalfont Centre for Epilepsy, Chalfont St Peter, United Kingdom
[14]Stichting Epilepsie Instellingen Nederland (SEIN), Heemstede, the Netherlands
* these authors are joint senior author

Running title: Iron metabolism in epilepsy

Corresponding author: Dr. E. Aronica
Amsterdam UMC, University of Amsterdam,
Dept. (Neuro)Pathology,
Meibergdreef 9, 1105 AZ, Amsterdam, the Netherlands
Phone: + 31 20 5664369
Email: e.aronica@amsterdamumc.nl

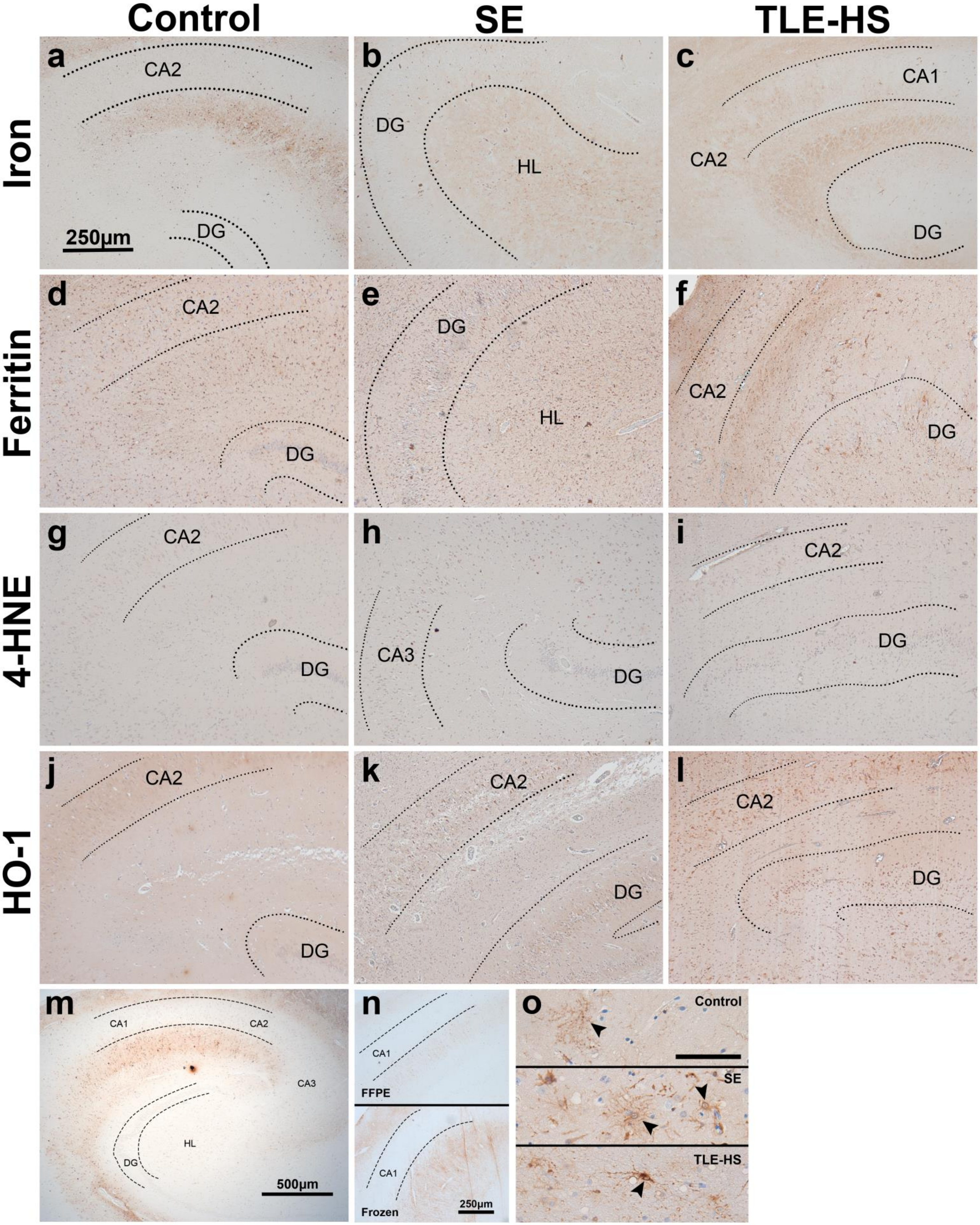

Control
SE
TLE-HS
Iron
Ferritin
4-HNE
HO-1
a
CA2
DG
250µm
b
DG
HL
c
CA1
CA2
DG
d
CA2
DG
e
DG
HL
f
CA2
DG
g
CA2
DG
h
CA3
DG
i
CA2
DG
j
CA2
DG
k
CA2
DG
l
CA2
DG
m
CA1
CA2
CA3
HL
DG
500µm
n
CA1
FFPE
CA1
Frozen
250µm
o
Control
SE
TLE-HS

Online resource 1 Low power overview iron, ferritin, 4-HNE and HO-1 immunohistochemistry in autopsy control, autopsy SE and surgical TLE-HS hippocampi (a-l) Representative low power micrographs from autopsy control, autopsy SE and surgical TLE-HS tissue. Iron staining in autopsy control hippocampus was most prominent in white matter-rich strata (*stratum oriens*/*stratum radiatum*/*stratum lacunosum moleculare*) and accumulated in oligodendrocytes. The cell-rich subfields cornu ammonis (CA), dentate gyrus (DG) and hilus (HL) hardly contained iron. In contrast, the hilus revealed more iron in the hilus in SE tissue, while TLE-HS hippocampi revealed additional iron accumulation in the CA1/CA2 region. Ferritin expression was seen predominantly in microglia and oligodendrocytes in control tissue, but was highly expressed in astrocytes in SE and TLE-HS tissue. 4-HNE and HO-1 expression was markedly elevated in CA1/CA2 neurons in SE and TLE-HS tissue as compared to autopsy control. **(m)** Low power overview of iron distribution in the whole hippocampus of an autopsy control case. **(n)** Comparison of iron staining in formalin-fixed paraffin-embedded (FFPE) tissue versus fresh frozen tissue of the same autopsy control case revealed lower intensity, but similar localization of iron. **(o)** Ferritin expression in the EC could be detected in fine astrocytic processes in control tissue, while SE and TLE-HS tissue displayed stronger ferritin expression in astrocytes. Sections d-l, o were counterstained with hematoxylin. Scale bars: 500 μm in m, 250 μm in a-l & n, 15 μm in o; arrowheads = glia.

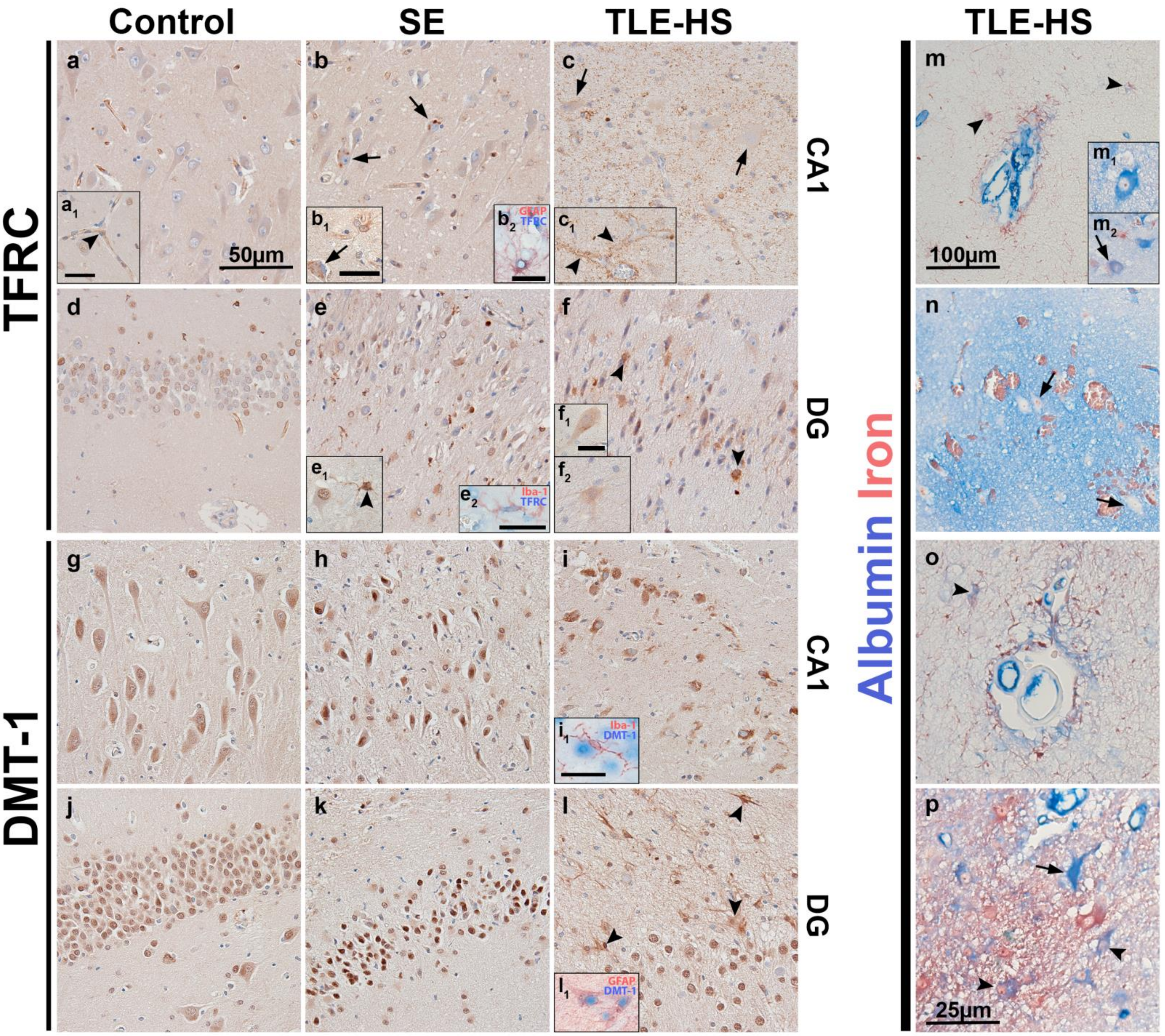

Online resource 2 Representative micrographs of TFRC, DMT-1 and iron/albumin immunohistochemistry in human autopsy control, autopsy SE and surgical TLE-HS hippocampus (a, d) TFRC staining in autopsy control tissue could be detected

predominantly in endothelial cells (a_1, arrowhead) and perinuclear in DG neurons. **(b, e)** SE tissue revealed TFRC positive inclusions in some CA1 (b, b_1, arrows) and some DG neurons (e). In addition, strong TFRC expression could be detected in microglia (e_2) and astrocytes (b_2, b_1, arrowhead). **(c, f)** TFRC expression in TLE-HS hippocampi revealed strong neuronal expression in CA1 and DG, especially in pyknotic cells (c, arrow, f_1). Astrocytes (c, arrowhead; f_2) and other glia (f, arrowheads) as well as astrocytic endfeet around microvessels (c_1, arrowheads) also displayed higher TFRC reactivity. **(g, j)** DMT-1 expression in autopsy control tissue was detected in neurons and glia, predominantly perinuclear but also in intracellular inclusions and the cell membrane. **(h, k)** SE tissue revealed stronger perinuclear staining in neurons, while glial staining was similar to control tissue. **(i, l)** TLE-HS tissue revealed strong DMT-1 reactivity in cell membranes of CA1 neurons while DG neurons appeared similar to control. Additionally, a markedly stronger expression could be detected in astrocytes (l, l_1, arrowheads) but not microglia (i_1). **(m-p)** Co-labeling of iron and albumin revealed co-localization of perivascular iron deposits with albumin in glial processes and neurons (m, m_1, m_2, o, p, arrowheads). Albumin reactivity was found in the neuropil (n) and occasionally inside neurons and glia (p). Sections a-l were counterstained with hematoxylin. Scale bars: 100 µm in m, 50 µm in a (representative for a-l, n, o), 25 µm in p, e_2, 20 µm in a_1 (representative for c_1), 15 µm in b_2 and i_1 (representative for l_1) and 10 µm in b_1 (representative for e_1, f_2, m_1, m_2); arrows = neurons, arrowheads = glia.

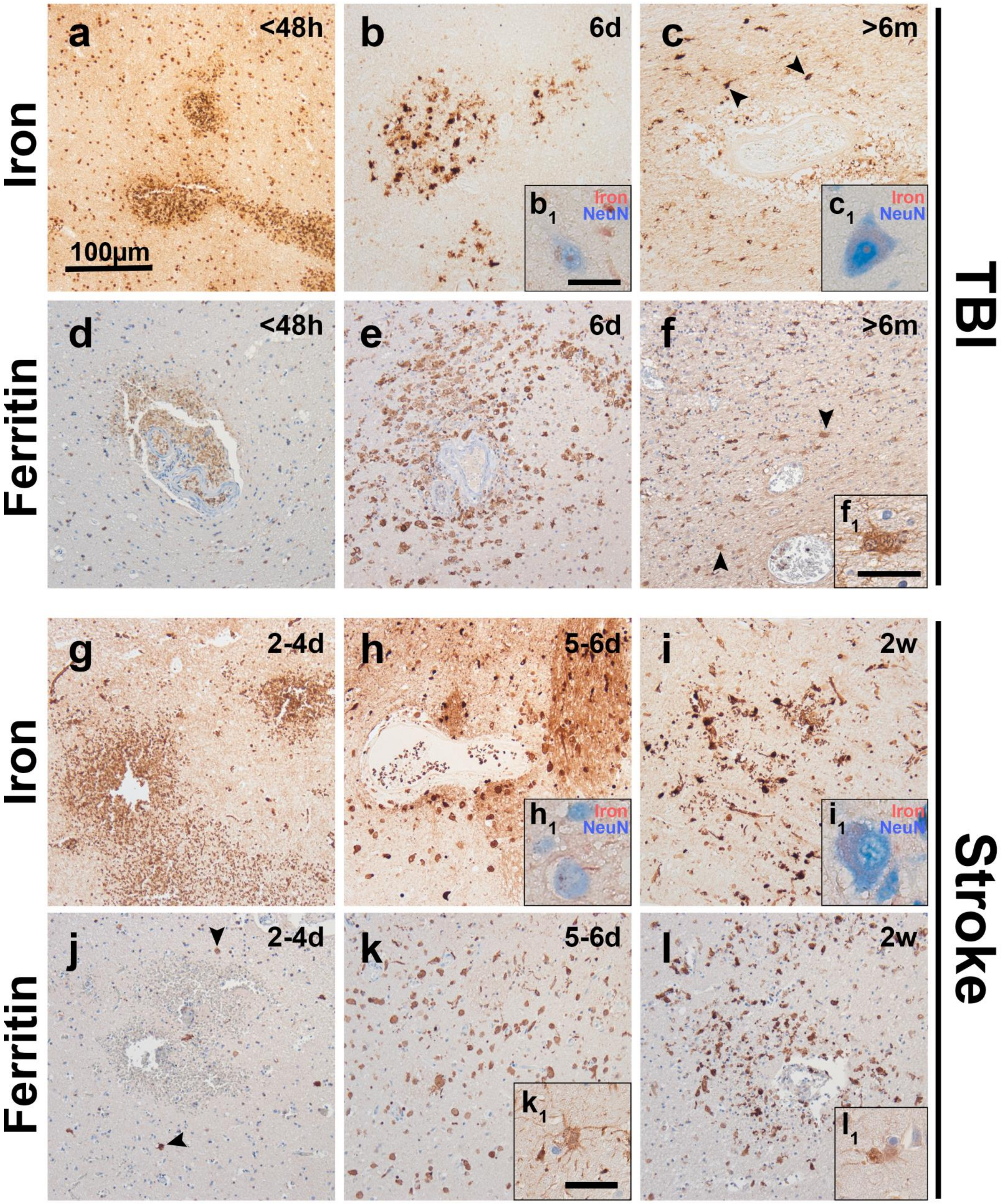

Iron
Ferritin
Iron
Ferritin
TBI
Stroke
a
<48h
100µm
b
6d
b_1
Iron
NeuN
c
>6m
c_1
Iron
NeuN
d
<48h
e
6d
f
>6m
f_1
g
2-4d
h
5-6d
h_1
Iron
NeuN
i
2w
i_1
Iron
NeuN
j
2-4d
k
5-6d
k_1
l
2w
l_1

Online resource 3 After trauma or stroke extravasation of iron from blood vessels and time-dependent iron accumulation in neurons and glia as well as astrocytic ferritin expression is evident (a, d) Acute lesions (<48 h *ante mortem*) due to brain trauma displayed leakage of iron-laden erythrocytes and enrichment of ferritin expressing cells around ruptured vessels. **(b, e)** Slightly older lesions (6 days *ante mortem*) revealed iron sequestration in glia and macrophages, iron accumulation in nucleolus and cytoplasm of peri-lesional neurons (b_1), as well as strong ferritin expression around damaged vessels. **(c, f)** Older lesions (>6 months *ante mortem*) from patients who developed epilepsy after TBI revealed overall higher iron accumulation (but more diffuse than in b) and ferritin expression around lesions. Moreover, peri-lesional neurons displayed iron accumulation (c_1) while ferritin expression could be detected in astrocytes (f_1, arrowheads). **(g, j)** Brain tissue from patients after acute strokes (2-4 days *ante mortem*) displayed extensive bleeding (g) and occasional ferritin expression by macrophages (j, arrowheads). **(h, i, k, l)** Older lesions (5 days-2 weeks *ante mortem*) displayed iron sequestration in reactive glia and macrophages as well as elevated ferritin expression around damaged vessels. Moreover, similar to TBI, older lesions displayed iron accumulation in peri-lesional neurons (h_1, i_1) and ferritin expression in astrocytes (k_1, l_1). Sections d-f, j-l were counterstained with hematoxylin. Scale bars: 100 µm in a (representative for a-l), 20 µm in f_1 and k_1 (representative for l_1), 10 µm in b_1 (representative for c_1, h_1, i_1). TBI: n = 2 (<48 h, 6 days) or 3 (>6 months with chronic epilepsy); Stroke: n = 3 (5-6 days, 2 weeks) or 4 (2-4 days).

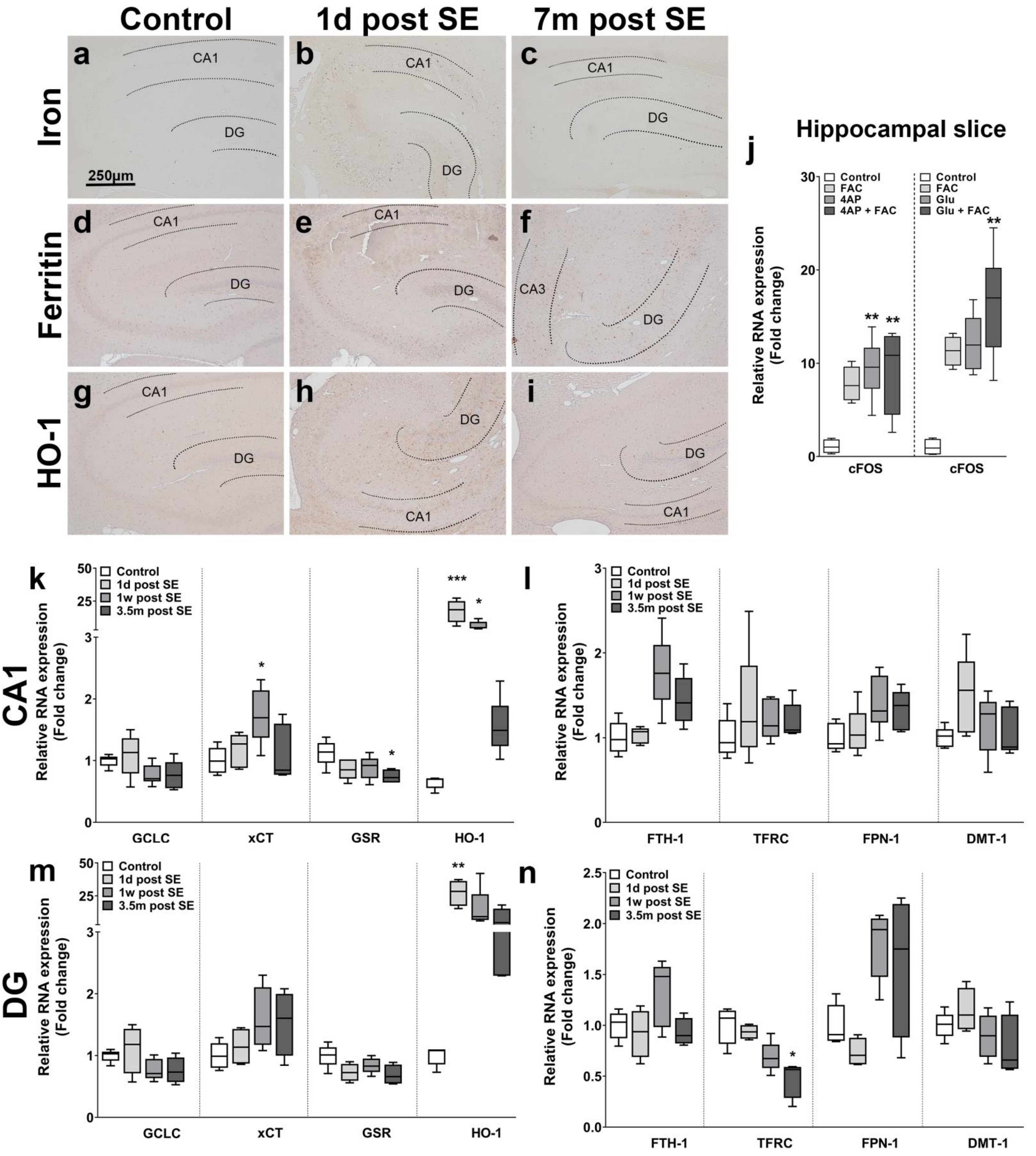

Control
1d post SE
7m post SE
Iron
Ferritin
HO-1
a
b
c
d
e
f
g
h
i
CA1
DG
CA3
250µm
j
Hippocampal slice
Control
FAC
4AP
4AP + FAC
Glu
Glu + FAC
Relative RNA expression (Fold change)
cFOS
k
CA1
Control
1d post SE
1w post SE
3.5m post SE
GCLC
xCT
GSR
HO-1
l
FTH-1
TFRC
FPN-1
DMT-1
m
DG
n

Online resource 4 Low power micrographs and RNA expression in hippocampal subregions of post-SE rat tissue reveals changes in glutathione and iron metabolism during the latent stage while acute hippocampal slice cultures upregulate cFOS RNA expression upon co-stimulation with ictogenic substances and iron (a-i) Low power micrographs of the hippocampus of post-SE rats stained for iron, ferritin and HO-1. Low iron accumulation, ferritin expression in microglia and low HO-1 expression in neurons could be found in control animals. 1 day post-SE iron strongly accumulated, while ferritin expression was detected in activated microglia and HO-1 in activated astrocytes. 7 months post-SE iron accumulated in glia and the neuropil, ferritin was expressed in microglia and astrocytes and HO-1 in neurons **(j)** RNA expression of cFOS in hippocampal brain slice preparations was elevated after 4-AP, 4-AP/FAC and Glu/FAC treatment. **(k, l)** RNA expression of HO-1 was strongly upregulated during the acute stage (1 day post-SE). In addition, HO-1 and xCT were upregulated during the latent stage (1 week post-SE), while GSR expression was reduced during the chronic stage (3.5 months post-SE). In contrast, iron metabolism genes were not different. **(m, n)** In the DG HO-1 was upregulated during the acute stage. Moreover, TFRC expression was downregulated during the chronic stage. Sections d-i were counterstained with hematoxylin. Scale bar: 250 µm in a (representative for a-i). j-n Kruskal-Wallis test followed by post-hoc Dunn's test. Data are expressed relative to expression observed in controls and displayed as Tukey box plot with whiskers representing range; $*p<0.05$, $**p<0.01$, $***p<0.001$. j: n = 6-7 slices from 3-4 animals per condition; k, l: n = 5 (control, 1 day post-SE, 3.5 months post-SE) or n= 6 (1 week post-SE) animals; m, n: n = 4 (1 day post-SE, 3.5 months post-SE) or n= 5 (control, 1 week post-SE) animals.

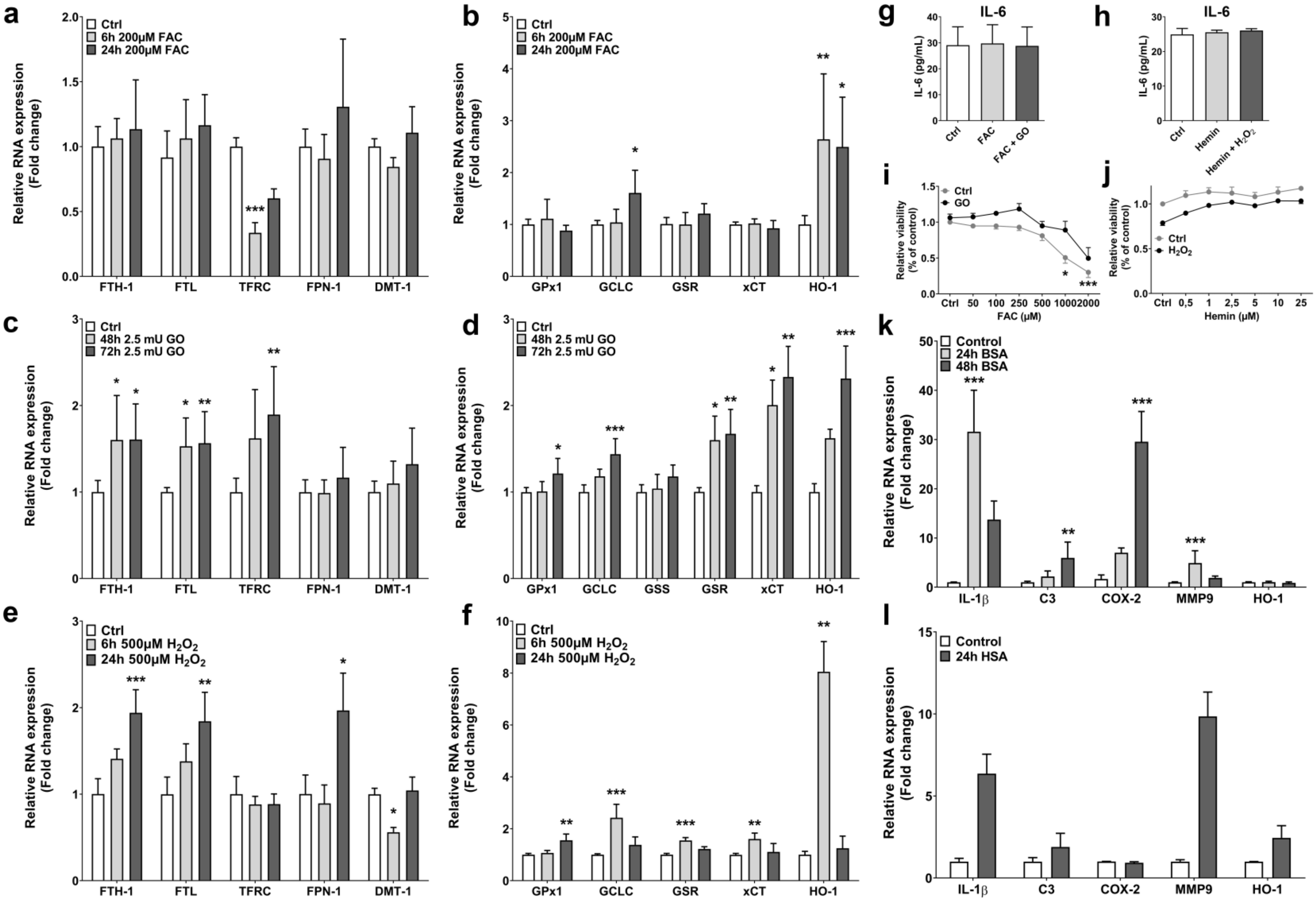

Online resource 5 Fetal astrocyte RNA expression, cytokine secretion and viability exposed to FAC, H_2O_2, GO, BSA or HSA (a, b) Fetal astrocytes exposed to 200 µM FAC showed lower expression of TFRC and higher expression of HO-1 after 6h and higher expression of GCLC and HO-1 after 24h incubation. **(c, d)** Stimulation of fetal astrocytes with chronic OS via 2.5 mU GO induced expression of GSR, xCT, FTH-1 and FTL after 48h. After 72h the same genes plus GPx1, GCLC, HO-1 and TFRC were induced. **(e, f)** Exposure to acute OS via 500 µM H_2O_2 induced antioxidant genes GCLC, GSR, xCT and

HO-1 and lowered DMT-1 expression after 6h, while after 24h GPx1, FTH-1, FTL and FPN-1 were elevated. **(g, h)** Secretion of IL-6 was not different in fetal astrocytes treated with FAC, GO, 10 μM hemin or a combination. **(k)** Stimulation of human fetal astrocytes with 300 μM BSA induced the expression of IL-1β and MMP9 after 24 h and C3 and COX-2 after 48 h. HO-1 expression was not changed. **(l)** Stimulation of fetal astrocytes with HSA for 24 h induced the expression of IL-1β and MMP9. Kruskal-Wallis test followed by post-hoc Dunn's test (a-h) or Two-way ANOVA followed by post-hoc Bonferroni's test (i, j). Data are expressed relative to expression observed in controls with SD (a-h) or SEM (i, j); *$p<0.05$, **$p<0.01$, ***$p<0.001$. n = 3 independent cultures in duplicates (a-f, k), n = 5 independent cultures (g, h), n = 2 independent cultures in quadruplicates (i), n = 2 independent cultures in triplicates (j), n = 2 cultures (l).

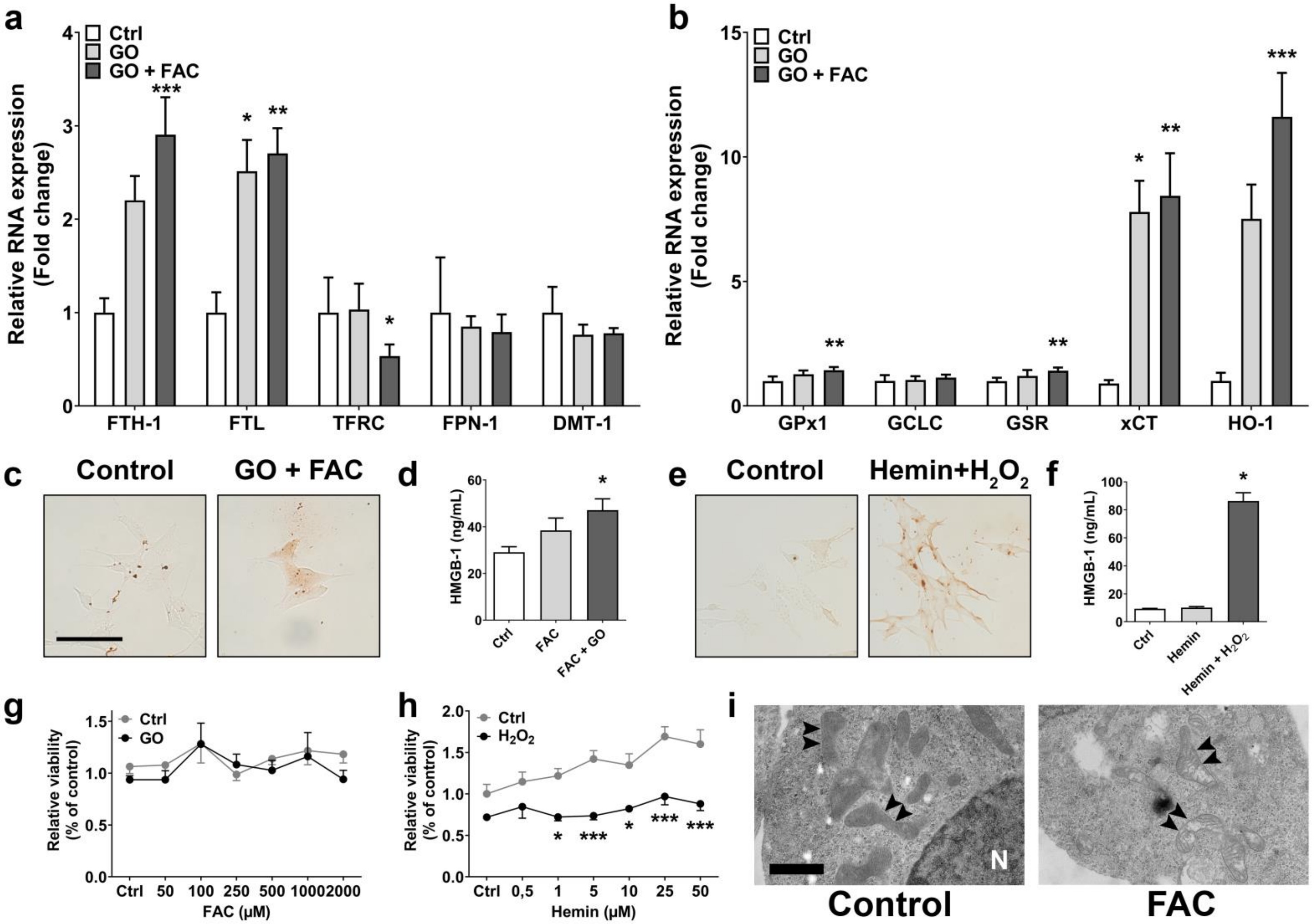

Online resource 6 SHSY5Y neuroblastoma cell RNA expression, iron accumulation, HMGB-1 secretion, viability and mitochondrial morphology upon exposure to FAC, GO or hemin (a, b) SHSY5Y neuroblastoma cells exposed to 2.5 mU GO for 48h display upregulation of xCT and FTL. Co-stimulation of SHSY5Y cells with GO plus 200 µM FAC induced the expression of GPx1, GSR, xCT, HO-1, FTH-1, FTL and reduced the expression of TFRC. **(c, d, g)** Small iron inclusions could be detected in untreated SHSY5Y cells while stimulation with FAC and GO for 24 h led to more cytoplasmic iron

accumulation (c). In addition, GO/FAC co-treatment induced secretion of HMGB-1 (d) while not affecting viability (g). **(e, f, h)** Stimulation of SHSY5Y cells with 10 µM hemin combined with 100 µM H_2O_2 induced iron uptake (e) and elevated HMGB-1 secretion into cell culture supernatant (f). Hemin treatment increased SHSY5Y cell viability, while co-treatment with H_2O_2 reduced viability. **(i)** Stimulation of SHSY5Y cells with FAC for 24h induced mitochondrial stress as indicated by reduced electron density (double arrowheads depict mitochondria; N = nucleus). Scale bar = 50 µm in c, 1 µm in i. Kruskal-Wallis test followed by post-hoc Dunn's test (a, b) or Two-way ANOVA followed by post-hoc Bonferroni's test (g, h). Data are expressed relative to expression observed in controls with SD (a, b) or SEM (g, h); *p<0.05, **p<0.01, ***p<0.001. n = 3 independent cultures in duplicates (a, b), n = 4 independent cultures (d, f), n = 2 independent cultures in quadruplicates (g) or n = 2 independent cultures in triplicates (h).

Online resource 7 Summary of clinical information of TBI and stroke cases

Pathology	Age	Gender	Area	Duration (injury to death)	Classification
TBI	75	m	SSC	<48 h	-
TBI	32	m	T	<48 h	-
TBI	34	m	F	6 days	-
TBI	65	f	F	6 days	-
TBI/epilepsy	35	m	-	6 months	-
TBI/epilepsy	52	m	EC	38 years	-
TBI/epilepsy	80	m	-	54 years	-
Stroke	81	f	EC	2 days	TACS
Stroke	52	f	F	2 days	TACS
Stroke	87	f	F	3 days	TACS
Stroke	42	f	P	3 days	TACS
Stroke	79	f	PUT	5 days	POCS + TACS
Stroke	85	f	PM	5 days	TACS
Stroke	55	m	O	6 days	TACS
Stroke	68	m	F	13 days	PACS
Stroke	78	f	P	14 days	TACS
Stroke	90	f	O	14 days	TACS

TBI, traumatic brain injury; m, male; f, female; SSC, somatosensory cortex; T, temporal; F, frontal; P, parietal; O, occipital; EC, entorhinal cortex; HC, hippocampus; PUT, putamen; PM, primary motor cortex; TACS, total anterior circulation infarct; PACS, partial anterior circulation infarct; POCS, posterior circulation infarct; h, hour

Online resource 8 Primer sequences used for quantitative real-time PCR

Species	Gene	Forward primer	Reverse primer
human	GPx1	TTCCCGTGCAACCAGTTT	GGACGTACTTGAGGGAATTCTG
	GCLC	AGTTGAGGCCAACATGCGAA	GTGAACCCAGGACAGCCTAA
	GSR	CGTGGAGGTGCTGAAGTTCTC	ATGGTCATGACTGGTAGCCT
	xCT	TGACTGGAGTCCCTGCGTAT	TCTTCTTCTGGTACAACTTCCAGT
	HO-1	GGCCAGCAACAAAGTGCAAG	AGTGTAAGGACCCATCGGAGA
	FTH-1	GTGCGCCAGAACTACCACCA	ACATCATCGCGGTCAAAGTAGT
	FTL	CTGGAGAAAAAGCTGAACCAG	TCCAGGAAGTCACAGAGATGG
	TFRC	GCGGCTGCAGGTTCTTCT	CATCTACTTGCCGAGCCAGG
	FPN-1	GTGGATCCTTGGCCGACTAC	AAGTGCCACATCCGATCTCC
	DMT-1	AGCTGGCATTGGGAAAGTCA	GGTGGATACCTGAGTGGCTG
	IL-1β	GCATCCAGCTACGAATCTCC	GAACCAGCATCTTCCTCAGC
	IL-6	CTCAGCCCTGAGAAAGGAGA	TTTCAGCCATCTTTGGAAGG
	C3	CCTGAAGATAGAGGGTGACCA	CCACCACGTCCCAGATCTTA
	MMP9	GAACCAATCTCACCGACAGG	GCCACCCGAGTGTAACCATA
	C1ORF43	GATTTCCCTGGGTTTCCAGT	ATTCGACTCTCCAGGGTTCA
	EF1α	ATCCACCTTTGGGTCGCTTT	CCGCAACTGTCTGTCTCATATCAC
Rat	GCLC	TTTGCACGATAACTTCATTTCC	CGTCTGGAAAGAAGAGGGACT
	xCT	TGTAACAGCTGTGGGCATCA	GGAAAATCTGGATCCGGGCA
	GSR	TTCCTCATGAGAACCAGATCC	CTGAAAGAACCCATCACTGGT
	HO-1	CAACCCCACCAAGTTCAAACA	AGGCGGTCTTAGCCTCCTCTG
	FTH-1	CGCCAGATCAACCTGGAGTT	GCAAAGTTCTTCAGGGCCAC
	TFRC	GTGCTTCAGAGTGCTCCCTTG	TCTCCATCTACTTGCCGAGC
	FPN-1	AGCCATCATTGGTGACTGGG	TCAGGATGATCCCGCAGAGA
	DMT-1	GCAGTGGTTAGCGTGGCTTA	TCTTCGCTCAGCAGGACTTT
	CycA	CCCACCGTGTTCTTCGACAT	AAACAGCTCGAAGCAGACGC
	GAPDH	ATGACTCTACCCACGGCAAG	TACTCAGCACCAGCATCACC
Mouse	FTH-1	CAGAACTACCACCAGGACGC	AGCCACATCATCTCGGTCAA
	FTL	CAGCCGCCTTTACAAGTCTC	TAGGAGCTAACCGCGAAGAG
	TFRC	GAGGCGCTTCCTAGTACTCC	CTTGCCGAGCAAGGCTAAAC
	HO-1	CCTCACAGATGGCGTCACTT	GCTGATCTGGGGTTTCCCTC
	IL-1β	TGAAGTTGACGGACCCCAAA	TGATGTGCTGCTGCGAGATT
	IL-6	GTTCTCTGGGAAATCGTGGA	TGTACTCCAGGTAGCTATGG
	cFOS	CAGCCTTTCCTACTACCATTCC	ACAGATCTGCGCAAAAGTCC
	TBP	GGAGAATCATGGACCAGAACA	GATGGGAATTCCAGGAGTCA
	HPRT	TCCTCCTCAGACCGCTTTT	CCTGGTTCATCATCGCTAATC
